# Decomposition of Halogenated Molybdenum Sulfide Dianions [Mo_3_S_7_X_6_]^2–^ (X = Cl, Br, I)

**DOI:** 10.1021/jasms.2c00162

**Published:** 2022-07-29

**Authors:** Marco Pritzi, Tobias F. Pascher, Marie-Luise Grutza, Philipp Kurz, Milan Ončák, Martin K. Beyer

**Affiliations:** † Institut für Ionenphysik und Angewandte Physik, Universität Innsbruck, Technikerstrasse 25, 6020 Innsbruck, Austria; ‡ Institut für Anorganische und Analytische Chemie, Albert-Ludwigs-Universität Freiburg, Albertstrasse 21, 79104 Freiburg, Germany

## Abstract

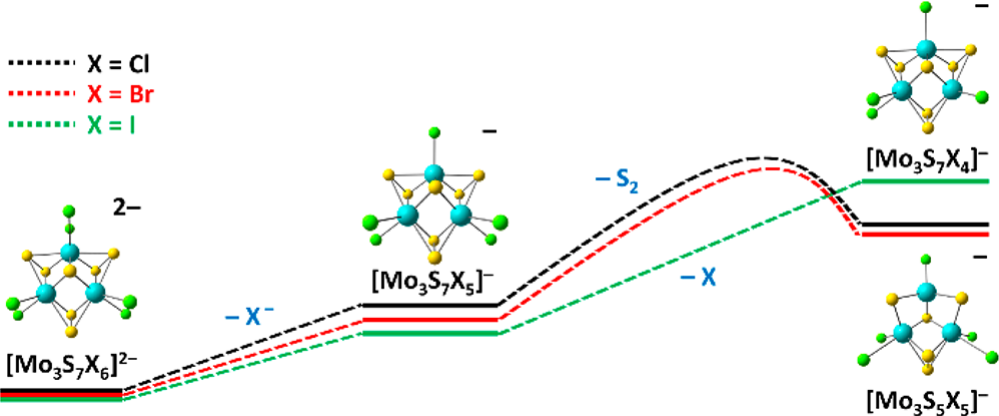

Molybdenum sulfides are considered a promising and inexpensive alternative to platinum as a catalyst for the hydrogen evolution reaction. In this study, we perform collision-induced dissociation experiments in the gas phase with the halogenated molybdenum sulfides [Mo_3_S_7_Cl_6_]^2–^, [Mo_3_S_7_Br_6_]^2–^, and [Mo_3_S_7_I_6_]^2–^. We show that the first fragmentation step for all three dianions is charge separation via loss of a halide ion. As a second step, further halogen loss competes with the dissociation of a disulfur molecule, whereas the former becomes energetically more favorable and the latter becomes less favorable from chlorine via bromine to iodine. We show that the leaving S_2_ group is composed of sulfur atoms from two bridging groups. These decomposition pathways differ drastically from the pure [Mo_3_S_13_]^2–^ clusters. The obtained insight into preferred dissociation pathways of molybdenum sulfides illustrate possible reaction pathways during the activation of these substances in a catalytic environment.

## Introduction

The transition to renewable energies entails several problems. The geographical and temporal fluctuation of most renewable energy sources needs to be overcome via storage solutions for a successful transition from fossil fuels to sustainable energy. One possibility for this is to produce hydrogen, which is storable and transportable, by electrolysis of water.^1^ Hydrogen produced using renewables such as solar or wind power is called green hydrogen, which plays a prominent role in power-to-gas schemes.^2^ The electrolysis of water consists of two half-reactions, the oxygen evolution reaction (OER), and the hydrogen evolution reaction (HER).^3,4^ To produce economically viable amounts of hydrogen, both reactions need to be efficient and therefore catalysts are needed. The HER under acidic conditions is best catalyzed by platinum.^5^ However, the scarcity and high price of platinum makes it economically unviable for large scale hydrogen evolution catalysis. Therefore, a goal of recent studies is to find a inexpensive and abundant material with a comparable catalytic activity toward hydrogen evolution.

A promising alternative for platinum are molybdenum sulfides such as MoS_2_.^6,7^ In 2012, Karunadasa et al. reported the synthesis of a well-defined molybdenum disulfide.^8^ After electrochemical reduction, it was possible to catalyze the HER in acidic solution.^8^ However, to make molybdenum-based catalysts economically viable, the number of active sites, namely the number of edge sites, needs to be increased. Kibsgaard et al. investigated submonolayers of [Mo_3_S_13_]^2–^ nanoclusters and reported excellent HER activity and stability in 2014. Furthermore, the synthesis of the studied [Mo_3_S_13_]^2–^ clusters follows a scalable route.^9^ The high catalytic activity of amorphous molybdenum sulfides MoS_2+*x*_, *x* < 1, has been reported by several groups.^10−12^ In 2016, Tran et al. showed that amorphous MoS_2+*x*_ prepared as nanoparticles or thin films consists of discrete [Mo_3_S_13_]^2–^ building blocks.^13^ Following these promising results, a series of different molecular molybdenum sulfide models have been synthesized by Wu and co-workers.^14−18^ Dave et al. for the first time reported high reactivity of [Mo_3_S_13_]^2–^ in homogeneous, visible light-driven HER in 2018.^19^ They highlighted the importance of the terminal disulfides in the catalytic process and found a higher reactivity if the terminal disulfides are partially replaced by water. However, the nanoclusters showed reduced catalytic activity if the terminal disulfides were replaced by halogenides to form [Mo_3_S_7_Cl_6_]^2–^ and [Mo_3_S_7_Br_6_]^2–^.^19^ An acid–base-resistant ligand-modified molybdenum sulfur cluster was recently reported by Zhang, Lin, and co-workers to exhibit enhanced photocatalytic HER activity.^20^

Gas-phase studies offer the advantage of dealing with well-defined and controllable systems. Such investigations give conceptual insight into the structure and behavior of individual molecules or small clusters as a model system for heterogeneous catalysis.^21−24^ Lang, Zhou, and Schwarz managed to obtain Fe_2_S_*x*_^+^ (*x* = 1–3) clusters and reacted them with small alkanes,^25^ which provided insight into the oxidative power of these species. Earlier, molybdenum chalcogenide cations were investigated in the Schwarz group.^26^ Intrinsic properties of molybdenum sulfide and oxide clusters were investigated by Jarrold, Raghavachari, and co-workers,^27−31^ including reactions with H_2_ and H_2_O.^29,32−36^ A reactivity experiment of small cationic molybdenum clusters with dimethyl disulfide in our group illustrated the high sulfur affinity of molybdenum.^37^

We recently used a bottom-up approach and studied the [Mo_3_S_13_]^2–^ nanocluster and its protonated forms [HMo_3_S_13_]^−^ and [H_3_Mo_3_S_13_]^+^ in the gas phase.^38^ Collision-induced dissociation (CID) experiments showed a variety of H_*x*_S_*y*_ elimination channels, and together with quantum chemical calculations it was shown that the Mo_3_ core stays intact. Furthermore, it was concluded that the studied clusters are structurally very flexible. The calculations suggested that the protonation primarily happens on the terminal disulfides, supporting a sulfur-centered HER mechanism. Infrared multiple photon dissociation spectroscopy of the [HMo_3_S_13_]^−^ cluster confirmed that the terminal disulfide is the protonation site.^39^ Protonation in the gas phase followed by Coulomb explosion was probed in the reactions of [Mo_2_O_2_S_6_]^2–^ and [Mo_2_O_2_S_5_]^2–^ with organic acids.^40^ Further insight was gained by the comparison with the fragmentation pathways of molybdenum oxide^41^ and oxysulfide^42^ clusters.

**Scheme 1:**
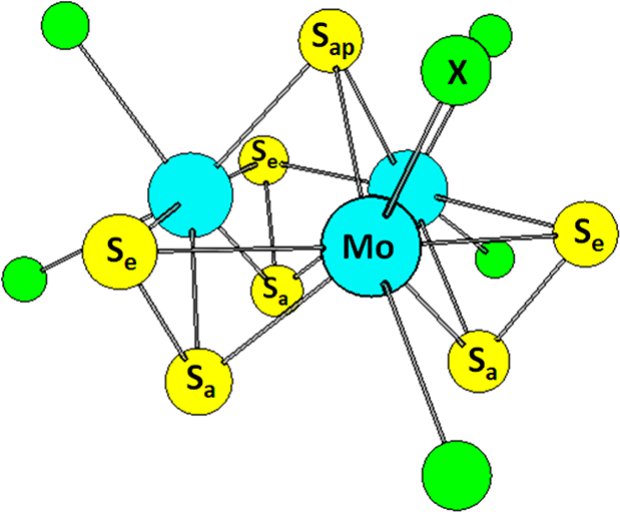
Three Types of Sulfur Atoms in [Mo_3_S_7_X_6_]^2–^: Equatorial (S_e_), Axial (S_a_), and Apical (S_ap_)

The halogenated molybdenum sulfides [Mo_3_S_7_Cl_6_]^2–^, [Mo_3_S_7_Br_6_]^2–^, and [Mo_3_S_7_I_6_]^2–^ result from replacing each terminal disulfide unit of [Mo_3_S_13_]^2–^ by two halide ions.^43^ These species are particularly useful for mechanistic studies, because only bridging disulfide units or apical sulfur atoms are available. Scheme 1 illustrates the structure for the [Mo_3_S_7_X_6_]^2–^compound. The [Mo_3_S_7_] core consists of an equilateral triangle of Mo atoms, which are bridged by disulfide units. In these, one S atom lies in the Mo_3_ plane and is denoted equatorial or S_e_, and the second sulfur atom is axial S_a_, following the nomenclature of Fedin et al.^43^ A seventh sulfur atom caps the Mo_3_ triangle on the opposite side of the S_a_ atoms and is referred to as apical sulfur atom, S_ap_.

Here we use CID to investigate the structural properties and rearrangement reactions of the gas-phase halogenated molybdenum sulfides [Mo_3_S_7_Cl_6_]^2–^, [Mo_3_S_7_Br_6_]^2–^, and [Mo_3_S_7_I_6_]^2–^. The study of these structures is interesting not only because of their changed HER activity but also because it could help to understand the decomposition and rearrangements of [Mo_3_S_13_]^2–^ during catalyst activation, building on our earlier CID study of [Mo_3_S_13_]^2–^.^38^

## Experimental and Computational Details

### Sample Preparation

Our sustained off-resonance irradiation CID (SORI-CID) experiments were performed with isotopically enriched [^92^Mo_3_S_7_Cl_6_]^2–^ and [^92^Mo_3_S_7_Br_6_]^2–^, as well as nonisotopically enriched [Mo_3_S_7_I_6_]^2–^. For the preparation of the halogenated clusters, a synthesis route described by Fedin et al. was followed.^43^ (NH_4_)_2_[^92^Mo_3_S_13_] was produced as described previously.^38,44^ To obtain [(CH_3_)_4_N]_2_[^92^Mo_3_S_7_Cl_6_], 150 mg of (NH_4_)_2_[^92^Mo_3_S_13_] was mixed with 15 mL of concentrated hydrochloric acid HCl (37%), heated for 20 min, and then filtered. The filtrate was mixed with 150 mg of tetramethylammonium chloride (CH_3_)_4_NCl and left in the refrigerator to crystallize. The product was then washed with water (2 × 8 mL), ethanol (6 mL), and diethyl ether (8 mL) and left to dry overnight under air. The obtained [(CH_3_)_4_N]_2_[^92^Mo_3_S_7_Cl_6_] is a red powder.

[(CH_3_)_4_N]_2_[^92^Mo_3_S_7_Br_6_] was synthesized in a very similar manner. 20.15 mg of (NH_4_)_2_[^92^Mo_3_S_13_] was mixed with 2 mL of concentrated hydrobromic acid HBr (48%), heated for 20 min, and filtered. The filtrate was mixed with 56 mg of tetramethylammonium bromide (CH_3_)_4_NBr and left in the refrigerator to crystallize. The product was washed in the same way and with the same amounts of water, ethanol, and diethyl ether as for the chlorinated compound described above. The product was obtained as an orange powder.

For the synthesis of [(CH_3_)_4_N]_2_[Mo_3_S_7_I_6_], about 10 times more starting material was required because of low yields. Because iodine is monoisotopic, experiments without isotopic enrichment were still manageable. Nonisotopically enriched (NH_4_)_2_[Mo_3_S_13_] was prepared as described above for the isotopically enriched version. Then 256 mg of (NH_4_)_2_[Mo_3_S_13_] was mixed with 25 mg of concentrated, nonstabilized hydroiodic acid HI (57%), heated for 20 min, and filtered. The filtrate was mixed with 250 mg of tetramethylammonium chloride (CH_3_)_4_NCl and left in the refrigerator to crystallize. The product was washed with 30 mL of water, 20 mL of ethanol, and 20 mL of diethyl ether. [(CH_3_)_4_N]_2_[Mo_3_S_7_I_6_] was obtained as a dark red powder.

### Experimental Setup and Measurements

CID experiments were performed on a Bruker Apex Qe FT-ICR mass spectrometer with Nanobay Console, equipped with a 9.4 T superconducting magnet. The ions are produced in a Dual Source II, a combined electrospray ionization/matrix-assisted laser desorption/ionization (ESI/MALDI) source.^45^ Typically, solutions of the samples at a concentration of about 0.3 mM in acetonitrile were used. After the ions are produced by ESI, they are transferred through multiple stages of radiofrequency and electrostatic ion guides into the ICR cell, where they were mass selected via sweep and shot isolation. Argon was used as a collision gas at a pressure regime of around 10^–8^ mbar. SORI-CID was performed by acquiring multiple mass spectra after off-resonance irradiation of the mass-selected ions with a fixed frequency offset of 500 Hz and a pulse length of 1 s, with increasing SORI power. Plotting the normalized intensities of reactant and fragment ions as a function of SORI power yields the fragmentation curve.

With the samples of the chlorinated molybdenum sulfide, we performed SORI-CID on [^92^Mo_3_S_7_Cl_6_]^2–^ and [^92^Mo_3_S_7_Cl_5_]^−^. With the brominated sample, we only studied [^92^Mo_3_S_7_Br_6_]^2–^ because this experiment already provided the relevant information. For the iodinated species, no isotopically enriched version was available. [Mo_3_S_7_I_6_]^2–^ has a broad natural isotope distribution (see Figure S1). We isolated the full peak group and performed a relatively crude SORI-CID experiment to identify the primary fragmentation pathway by SORI at *m*/*z* 636.847 with a SORI power of 1.5%. This allowed us to identify the preferred dissociation to I^–^ and [Mo_3_S_7_I_5_]^−^ (Figures S2 and S3). This dissociation pathway is very efficient, which leads to overall low signal levels of [Mo_3_S_7_I_6_]^2–^ due to dissociation events in the ion source region.

However, [Mo_3_S_7_I_5_]^−^ was obtained from the ESI source in high yields, and the SORI CID breakdown diagram of this species was obtained with decent signal-to-noise level. In this case, the peak at *m*/*z* 1148 was isolated. A simulation of an ultrahigh resolution mass spectrum with the different combinations of stable molybdenum and sulfur isotopes^46^ yields 91 isotopologues of [Mo_3_S_7_I_5_]^−^, with their exact mass ranging from *m*/*z* 1148.0253 to 1148.0466. This complexity is largely due to the isotopes of molybdenum. Fortuitously, fragmentation of [Mo_3_S_7_I_5_]^−^ does not involve loss of molybdenum atoms, while loss channels involving multiple iodine atoms or molecules dominate, and iodine is monoisotopic. For the observed disulfide loss, only the contribution of ^32^S_2_ with *m*/*z* 63.944 was considered, disregarding the minor contributions of, for example, ^32^S^33^S and ^32^S^34^S.

### Computational Details

To interpret the experimental results, quantum chemical calculations were performed at the B3LYP/def2TZVP level of theory using the D3 dispersion as suggested by Grimme et al.^47^ The obtained energetics are benchmarked against the ωB97XD/def2TZVP method in the Supporting Information (see Table S1). For the most relevant ions observed in the experiment, the structure with the lowest (zero-point corrected) energy was searched by optimizing different arrangements of molybdenum clusters in the lowest spin multiplicity. Minima are verified via frequency calculations while the stability of the wave function was tested prior to each optimization. Transition states (TS) are verified via intrinsic reaction coordinate (IRC) calculations or by applying a minor offset along the normal vector of the corresponding imaginary frequency in the TS followed by optimization. All calculations are zero-point energy (ZPE) corrected and were performed employing the Gaussian 16 package.^48^

## Results and Discussion

We investigated the decomposition pathways of the three halogenated molybdenum sulfides [Mo_3_S_7_Cl_6_]^2–^, [Mo_3_S_7_Br_6_]^2–^, and [Mo_3_S_7_I_6_]^2–^. For better readability, we omit the isotope label of ^92^Mo. An overview of all observed reactions is provided in Table 1.

**Table 1:** Overview of Fragmentation Channels for [Mo_3_S_7_X_6_]^2–^, X = Cl, Br, I, along with Suggested Neutral Counterparts^a^

halide X	reactants	products	abundance
X = Cl, Br, I	[Mo_3_S_7_X_6_]^2–^	[Mo_3_S_7_X_5_]^−^ + X^–^	high
X = Cl, Br	[Mo_3_S_7_X_5_]^−^	[Mo_3_S_5_X_5_]^−^ + S_2_	high
X = Cl	[Mo_3_S_5_Cl_5_]^−^	[Mo_2_S_4_Cl_2_]^−^ + MoSCl_3_	high
		[Mo_2_S_3_Cl_3_]^−^ + MoS_2_Cl_2_	high
		[Mo_2_S_3_Cl]^−^ + MoS_2_Cl_4_	high
		[Mo_2_S_2_Cl]^−^ + MoS_3_Cl_4_	low
X = Br	[Mo_3_S_5_Br_5_]^−^	[Mo_3_S_5_Br_4_]^−^ + Br	low
		[Mo_3_S_5_Br_3_]^−^ + Br_2_	low
		[Mo_3_S_5_Br_2_]^−^ + Br_2_ + Br	low
		[Mo_3_S_4_Br_4_]^−^ + SBr	low
		[Mo_2_S_4_Br]^−^ + MoSBr_4_	low
		[Mo_2_S_3_Br_3_]^−^ + MoS_2_Br_2_	low
		[MoS_3_Br]^−^ + Mo_2_S_2_Br_4_	low
		[MoSBr_3_]^−^ + Mo_2_S_4_Br_2_	low
X = I	[Mo_3_S_7_I_5_]^−^	[Mo_3_S_7_I_4_]^−^ + I	high
		[Mo_3_S_7_I_2_]^−^ + I_2_ + I	low
		[Mo_3_S_7_I]^−^ + 2 I_2_	low
		[Mo_3_S_7_]^−^ + 2 I_2_ + I	high
		[Mo_3_S_5_I_3_]^−^ + S_2_ + I_2_	low
		[Mo_3_S_5_I_2_]^−^ + S_2_ + I_2_ + I	low

a Neutral fragments Mo_*m*_S_*n*_X_*o*_ are given as stoichiometric sum and may have fragmented further.

### CID of [Mo_3_S_7_Cl_6_]^2–^ and [Mo_3_S_7_Cl_5_]^−^

**Figure 1:**
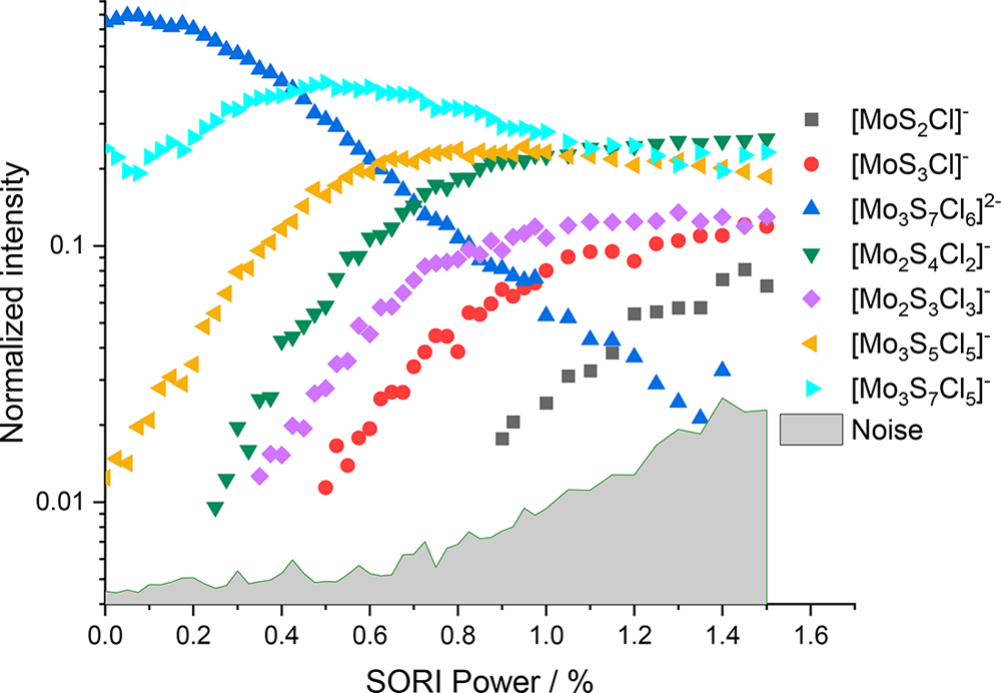
Fragmentation curve obtained from the SORI-CID experiment of [Mo_3_S_7_Cl_6_]^2–^.

The fragmentation curve of the chlorinated molybdenum sulfide dianion [Mo_3_S_7_Cl_6_]^2–^ shows a large onset (∼20%) of [Mo_3_S_7_Cl_5_]^−^, which corresponds to a Cl^–^ loss, shown in Figure 1. This suggests that it is energetically feasible to remove a chloride ion from the precursor. However, the [Mo_3_S_7_Cl_5_]^−^ in the fragmentation curve only rises to a SORI power of around 0.5%, after which it declines. Thus, the next fragment is [Mo_3_S_5_Cl_5_]^−^, most likely originating from [Mo_3_S_7_Cl_5_]^−^ via loss of S_2_. Four more fragments appear with increasing SORI power, [Mo_2_S_4_Cl_2_]^−^, [Mo_2_S_3_Cl_3_]^−^, [MoS_3_Cl]^−^, and [MoS_2_Cl]^−^. It is interesting to note that these four fragments involve loss of molybdenum atoms, i.e., the Mo_3_ subunit is destroyed. To verify that none of these products is a direct fragment of [Mo_3_S_7_Cl_6_]^2–^, we also performed a SORI-CID experiment using [Mo_3_S_7_Cl_5_]^−^ as a precursor.

Interestingly, Cl^–^ does not appear in the mass spectrum of the [Mo_3_S_7_Cl_6_]^2–^ fragmentation, although the corresponding [Mo_3_S_7_Cl_5_]^−^ is prominent. This can be explained by the kinetic energy release during Coulomb explosion: in dissociation of [Mo_3_S_7_Cl_6_]^2–^ into [Mo_3_S_7_Cl_5_]^−^ and Cl^–^, the two negatively charged ions undergo Coulomb explosion.^40,49,50^ Due to conservation of momentum, the lighter partner, in this case Cl^–^, carries the major fraction of the Coulomb energy as kinetic energy and can escape from the trapping potential. The alternative pathway, electron detachment followed by loss of a neutral Cl atom, is not plausible at low SORI power because the electron affinity of Cl is 3.61 eV.^51^

**Figure 2:**
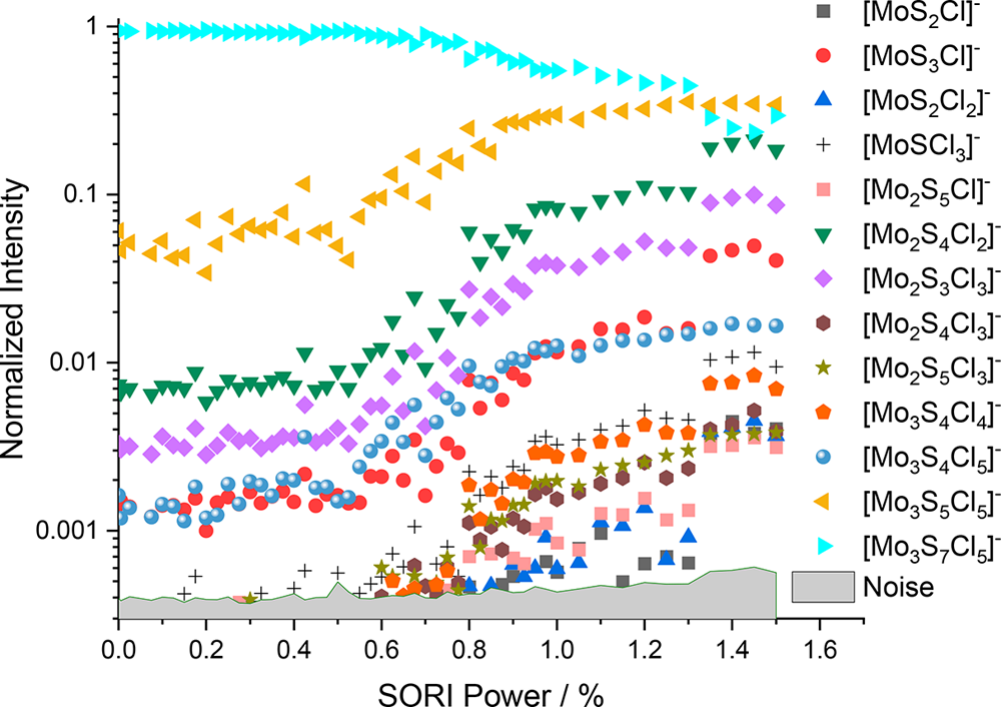
Fragmentation curve obtained from the SORI-CID experiment of [Mo_3_S_7_Cl_5_]^−^.

The CID experiment of [Mo_3_S_7_Cl_5_]^−^ is shown in Figure 2. The first fragment that arises is [Mo_3_S_5_Cl_5_]^−^, confirming sequential fragmentation. Overall, the results of this experiment illustrate a few challenging aspects: The intensity of multiple structures is initially in the % or ‰ range and stagnates up until a SORI power of around 0.5%, pointing toward residual kinetic excitation of a small fraction of ions during mass selection. Above 0.5%, many different fragments arise. Near the end of the fragmentation curve, there is a jump in the intensity of most fragments, hinting toward problems in maintaining a constant pressure during the measurements. Nevertheless, [Mo_3_S_5_Cl_5_]^−^, [Mo_2_S_4_Cl_2_]^−^, [Mo_2_S_3_Cl_3_]^−^, and [MoS_3_Cl]^−^ fragments appear in the same order as in the previous experiment, suggesting that direct fragmentation of [Mo_3_S_7_Cl_6_]^2–^ into one of those fragments did not take place. However, an additional fragment, [Mo_3_S_4_Cl_5_]^−^, is observed that was missing previously. On the other hand, the intensity of the [MoS_2_Cl]^−^ ion, which could be seen very well in the experiment with [Mo_3_S_7_Cl_6_]^2–^, is barely above the noise level in the present experiment and lies well below other fragments, which have not been seen before. These two observations suggest that a small fraction of the dissociation events take different routes in the two experiments.

### CID of [Mo_3_S_7_Br_6_]^2–^

**Figure 3:**
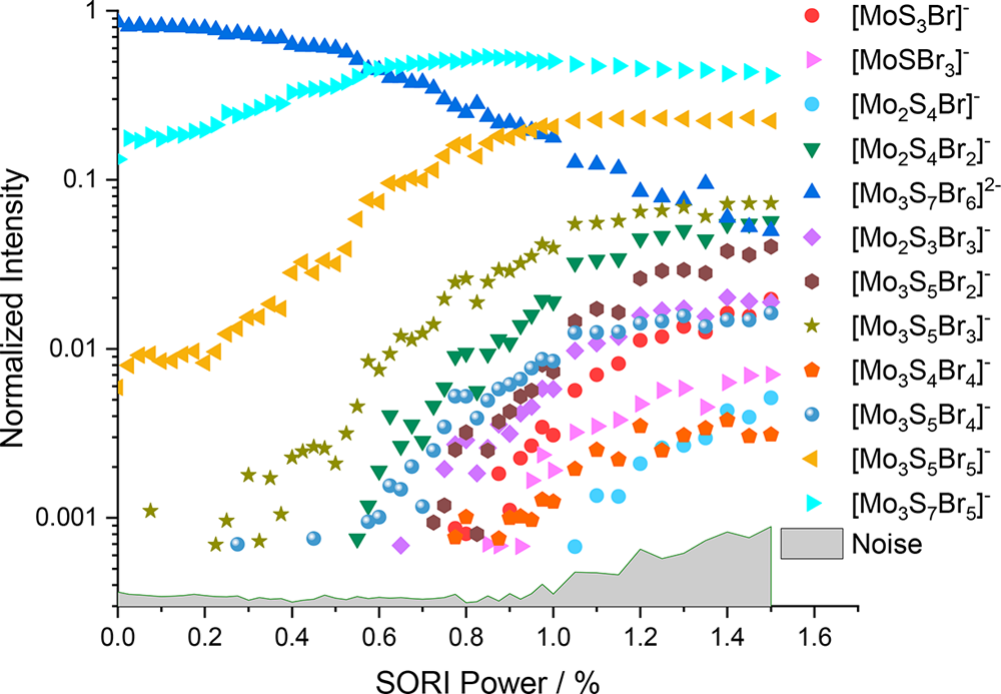
Fragmentation curve obtained from the SORI-CID experiment of [Mo_3_S_7_Br_6_]^2–^.

The fragmentation curve of the brominated molybdenum sulfide [Mo_3_S_7_Br_6_]^2–^, Figure 3, shows a very similar pattern as the chlorinated species. The first and most prominent fragmentation is the loss of Br^–^, leading to [Mo_3_S_7_Br_5_]^−^, which is the dominant fragment at all energies. Subsequent S_2_ loss leads to the next fragment, [Mo_3_S_5_Br_5_]^−^. In contrast to [Mo_3_S_7_Cl_6_]^2–^, the brominated structure shows fragmentation paths where the number of molybdenum atoms is mostly preserved. For example, the next most prominent fragment is [Mo_3_S_5_Br_3_]^−^, corresponding to a sequential Br_2_ loss. At SORI powers beyond 0.5%, a number of other fragments arise. Interestingly, the bromide ion is now seen in the mass spectrum, with its intensity rising with increasing SORI power. The ion is not shown in the figure, as its intensity is still compromised by the high kinetic energy release during Coulomb explosion, which explains why Br^–^ appears consistently with an intensity lower than that of the corresponding fragment [Mo_3_S_7_Br_5_]^−^. A fraction of the bromide ions probably still escapes from the trapping potential.

### CID of [Mo_3_S_7_I_6_]^2–^ and [Mo_3_S_7_I_5_]^−^

The SORI-CID experiment of [Mo_3_S_7_I_6_]^2–^ was more difficult, as no isotopically enriched sample was available. The seven natural isotopes of molybdenum lead to a complex isotope pattern. In addition, I^–^ loss is very facile, making ESI of the intact compound challenging. An example of the [Mo_3_S_7_I_6_]^2–^ peak group in the mass spectrum can be seen in Figure S1. More than 20 different peaks can be identified in this case, and each of these peaks is composed of numerous isotopologues, with more than 1000 isotopologues for the peak group in total. In contrast to bromine or chlorine, however, iodine is monoisotopic, which makes the experiment still feasible.

Because of the isotopic distribution and the overall low intensities of [Mo_3_S_7_I_6_]^2–^, we were only able to perform a crude SORI-CID experiment by acquiring 200 mass spectra with a SORI power of 0% and 1.5%, irradiating at *m*/*z* 636.847. We managed to identify [Mo_3_S_7_I_5_]^−^ together with I^–^ as the dominant fragments of [Mo_3_S_7_I_6_]^2–^. Again, we see the loss of a halogenide ion as the first fragmentation step. Already at 0% SORI power, the iodide ion can be seen in the mass spectrum (see Figure S2) whereas [Mo_3_S_7_I_5_]^−^ is missing. At a SORI power of 1.5% (Figure S3), the I^–^ peak is higher than the peaks of [Mo_3_S_7_I_5_]^−^. Because I^–^ is monoisotopic, all the intensity adds up in one peak while the [Mo_3_S_7_I_5_]^−^ fragment intensity is distributed over several peaks, explaining this difference. Again, iodine is heavier than bromine and chlorine and does not as easily escape from the trapping potential after Coulomb explosion.

**Figure 4:**
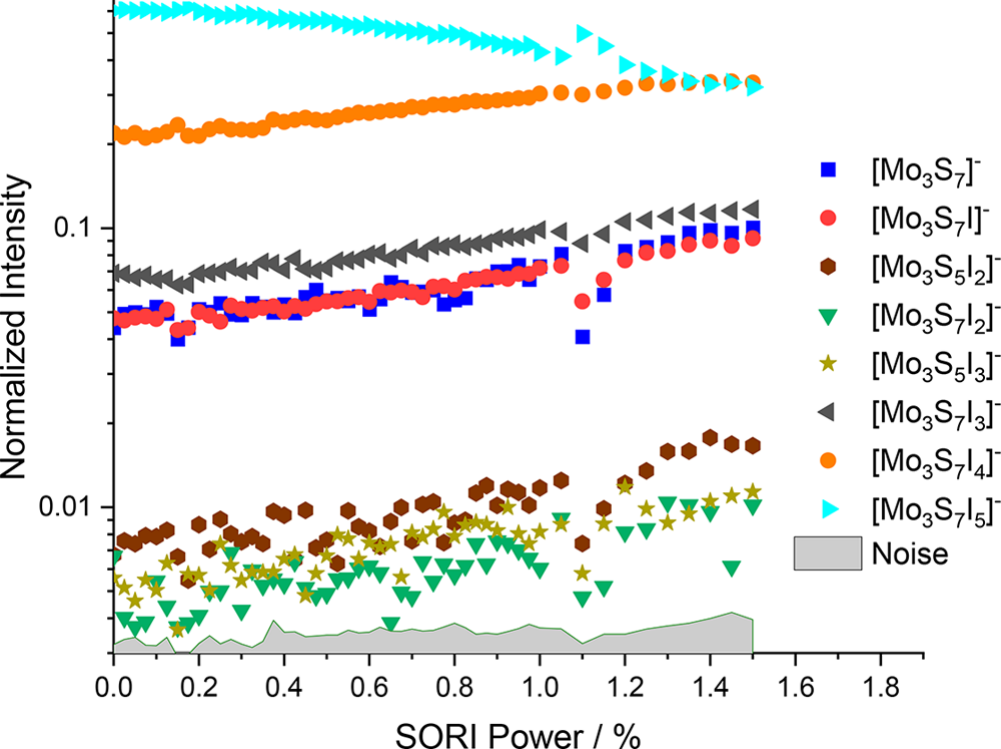
Fragmentation curve obtained from the SORI-CID experiment of [Mo_3_S_7_I_5_]^−^.

Having confirmed the first fragmentation step as I^–^ loss, we used [Mo_3_S_7_I_5_]^−^ as the precursor for a scanned SORI-CID experiment. Because of the many isotopes and the resulting low intensity, we chose very soft isolation parameters. For the analysis, we added up the intensities of all the isotopologues of a specific ion. For disulfur loss, only loss of ^32^S_2_ is considered, which accounts for 90% of S_2_ loss products. The fragmentation curve obtained can be seen in Figure 4. Unlike with the other halogenated molybdenum sulfides, loss of S_2_ to form [Mo_3_S_5_I_5_]^−^ was not observed. In addition, all observed fragments up to a SORI power of 1.5% retained all three molybdenum atoms. Interestingly, the fragments come in three intensity ranges. The loss of an iodine atom leading to [Mo_3_S_7_I_4_]^−^ is the most prominent fragmentation path. The fragments in the medium intensity range, consisting of [Mo_3_S_7_I_3_]^−^, [Mo_3_S_7_I]^−^, and [Mo_3_S_7_]^−^, cover loss of multiple I or I_2_ units, up to the elimination of all iodine atoms from the cluster. The [Mo_3_S_7_]^−^ fragment is particularly interesting, because five electrons have been transferred from the I^–^ ligand upon leaving as I atoms or I_2_ molecules, underlining the high redox capacity of the Mo_3_S_7_ core structure. In the low-intensity range, there are structures involving loss of disulfur units. The three ions observed at low intensities are [Mo_3_S_5_I_2_]^−^, [Mo_3_S_5_I_3_]^−^, and [Mo_3_S_7_I_2_]^−^.

### Computational Results

**Figure 5:**
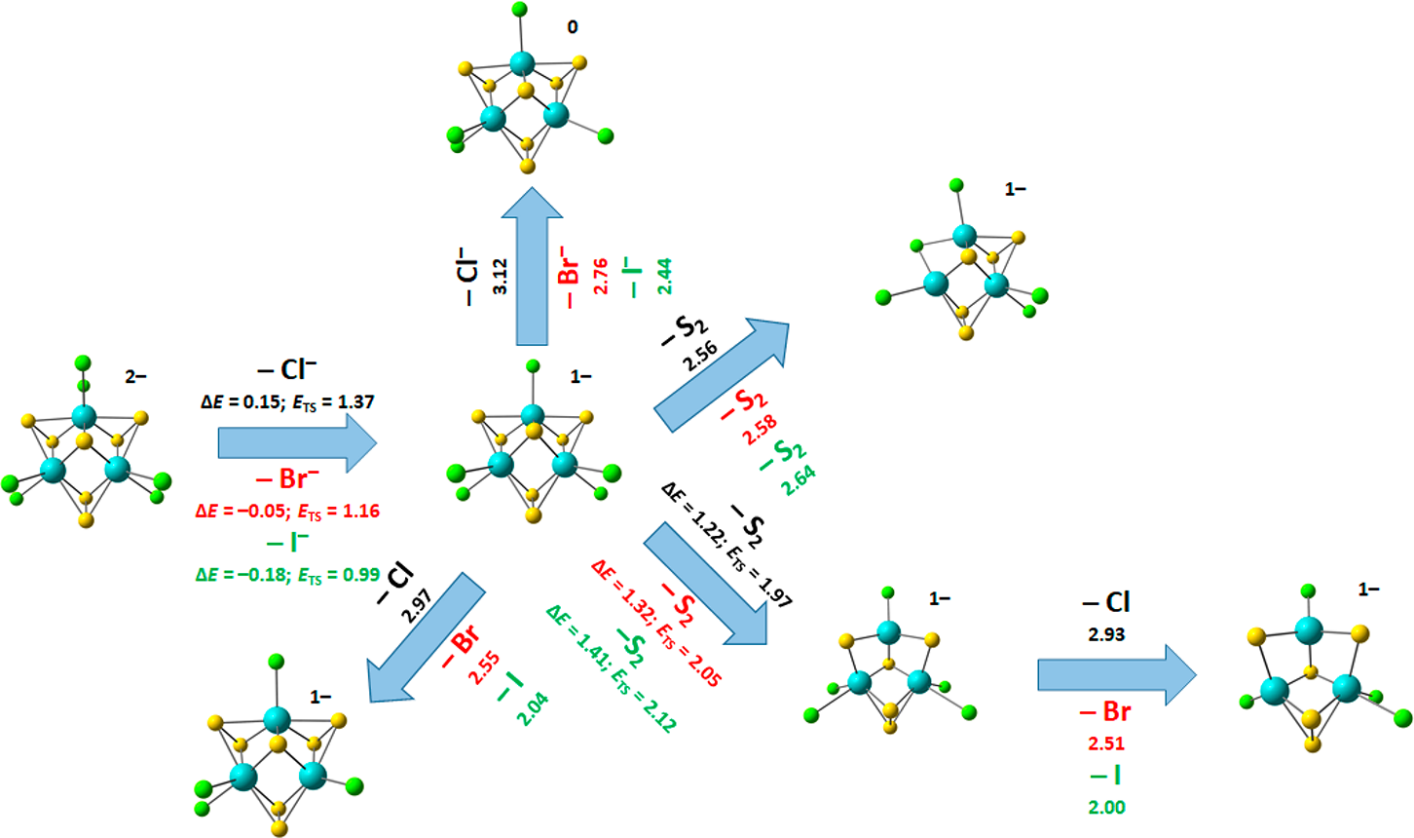
Quantum chemical calculations for different fragmentation pathways of [Mo_3_S_7_X_6_]^2–^, X = Cl, Br, I (black, red, and green text color, respectively). Reaction energies are shown in eV; activation energies for transition states *E*_TS_ are added where appropriate. Structures calculated at the B3LYP+D3/def2TZVP level of theory. Atom color code: Mo, blue; S, yellow; X, green.

**Figure 6:**
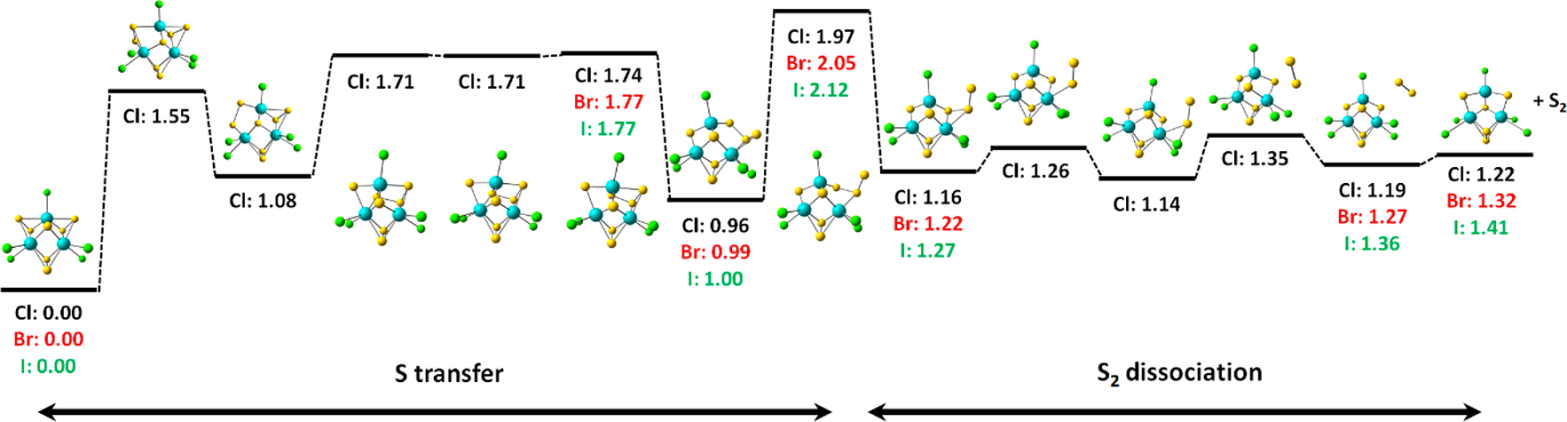
Reaction pathway for elimination of S_2_ from [Mo_3_S_7_X_5_]^−^, X = Cl. Calculated at the B3LYP+D3/def2TZVP level of theory, all energies are given in eV. For X = Br, I, the most important points are shown. Atom color code: Mo, blue; S, yellow; X, green.

To obtain insight into the cluster chemistry, we performed quantum chemical calculations of prominent dissociation pathways as summarized in Figures 5 and 6, which are potentially representative of the rearrangements taking place in a catalytic environment. The onset of the first fragment, [Mo_3_S_7_Cl_5_]^−^, can easily be explained (see Figure 5). The dissociation of the chloride ion is slightly endothermic and only stabilized by a Coulomb barrier of 1.37 eV. The endothermicity is expected to be sensitive to the basis set size, as was the case for the molybdenum oxysulfides.^40^ For the brominated molybdenum sulfide [Mo_3_S_7_Br_6_]^2–^, the dissociation of the halogenide ion is exothermic with −0.05 eV along with a Coulomb barrier of 1.16 eV, again explaining the large initial intensity in the experiment. This trend continues and the halogenide ion loss for [Mo_3_S_7_I_6_]^2–^ is even more exothermic at −0.18 eV, with a lower Coulomb barrier of 0.99 V. This, in addition to the scattering of the peak in the mass spectrum due to the isotopes, explains the very low intensity of [Mo_3_S_7_I_6_]^2–^ in the mass spectrum. It is quite intriguing that the kinetic energy release KER = *E*_TS_ – Δ*E* is almost identical for the three species, ranging from 1.17 to 1.22 eV.

After initial halogenide ion dissociates, there are multiple channels available, with the loss of S_2_ and the loss of a halogen atom being the most relevant (see Figure 5). Loss of another halogenide ion is unfavorable, and retaining the negative charge at the [Mo_3_S_7_X_4_] moiety is favored by 0.15–0.40 eV. For the S_2_ loss, two different pathways are plausible. The first is the direct loss of a bridging S_2_ unit, which is replaced by a bridging halogen atom at a cost of about 2.6 eV. However, a second pathway toward S_2_ loss, which requires prior transfer of one sulfur atom between two separate bridging units, is more plausible from both thermochemical and kinetic perspectives, with a barrier lying at 0.5–0.6 eV below the dissociation limit for the direct S_2_ loss channel.

The detailed reaction pathway of S_2_ loss from [Mo_3_S_7_X_5_]^−^ is shown in Figure 6. In the first step, the S–S bond of a bridging disulfide unit is weakened, leading to a short Mo–S bond from the equatorial sulfur atom to the molybdenum atom that has a vacant coordination site due to the previous loss of a halide ion. In the next step, the S–S bond breaks, and the S atom starts interacting with the neighboring bridging disulfide unit. A deep local minimum is reached with a bridging S_3_ unit that distorts the cluster structure. The rate-limiting transition state, lying at 1.97–2.12 eV, prepares for the elimination of a S_2_ unit, with the initially transferred S atom taking a stable bridging position. In the local minimum reached after this TS, the S_2_ unit is only weakly bound, and its final elimination requires the passage of several low-lying transition states. Note that the original bridging S_2_ group leaves and the transferred S atom fills the gap, avoiding undercoordinated molybdenum centers. However, it is quite probable that pathways exist which allow for scrambling of the sulfur atoms before the final S_2_ loss.

Due to the structural flexibility of molybdenum sulfide, which leads to a large number of possible rearrangements, we calculated the full reaction path only for X = Cl. For X = Br, I, the most relevant stationary points were located, including the rate-limiting transition state and the local minima it connects. The energy required for S_2_ loss increases with decreasing halogen electron affinity from chlorine to iodine, while the loss of a halogen atom becomes less endothermic. At the same time, direct dissociation of a halogen atom is entropically favored over the S_2_ dissociation pathway shown in Figure 6, which involves a series of tight transition states; dissociation of an iodine atom from [Mo_3_S_7_I_5_]^−^ with 2.04 eV lies below the barrier for S_2_ dissociation at 2.12 eV. While this small difference lies within the computational uncertainties, both entropy and calculated energetics are in line with the experimental observation of exclusive iodine loss.

### Discussion

The experiments also yield further insight into the stability of the Mo_3_ core unit. After the loss of S_2_ as the second step, the required energy for halogen atom loss remains relatively unchanged (see Figure 5). The pathways listed in Table 1 show that further fragmentation of the X = Cl species involves disintegration of the Mo_3_S_5_ core and loss of [Mo_*m*_S_*n*_Cl_*p*_] species, consistent with the high binding energy of chlorine. In contrast, the much weaker bound iodine atoms are readily lost as I or I_2_ units, while S_2_ elimination is observed in overall low abundance and most likely only after additional iodine loss. The strength of the Mo–X bond decreases from Mo–Cl through Mo–Br to Mo–I, and consistently, loss of [Mo_*m*_S_*n*_Br_*p*_] as well as Br, Br_2_, and even SBr is observed.

In our earlier study on the decomposition of [Mo_3_S_13_]^2–^ in the gas phase, we have shown that the initial step is S_2_ loss.^38^ From the thermochemical perspective, it was shown that in the most feasible dissociation reaction, S_2_ is formed from sulfur atoms from two bridging groups. Through introduction of halogen anions in the present study, we were able to pin down the mechanism for S_2_ elimination in [Mo_3_S_7_X_6_]^2–^, X = Cl, Br, and show that bridging S_2_ units are able to transfer a S atom to the adjacent Mo center. This makes it plausible that two adjacent bridging S_2_ groups transfer a S atom to the Mo center. The following scenarios for S_2_ loss from [Mo_3_S_13_]^2–^ are conceivable: (1) the initial terminal S_2_ is lost, followed by its reconstruction from two bridging S_2_ units; (2) two bridging S_2_ units transfer a S atom each to an adjacent Mo center, leading to two end-on bound S_2_ ligands; one S_2_ ligand is lost, while the other resumes the original side-on coordination of a terminal S_2_ ligand; (3) a S atom is transferred from a bridging S_2_ unit to Mo, transforming a terminal S_2_ unit to S_3_; S_2_ is eliminated from the S_3_ unit; a second S atom transfer from bridging S_2_ allows for the reconstruction of a terminal S_2_.

The S_2_ elimination from [Mo_3_S_7_X_5_]^−^ bears some parallels to the reaction of [Mo_3_S_7_X_6_]^2–^ with phosphines.^43^ Both are only observed for X = Cl, Br, and both involve the loss of equatorial sulfur atoms in the initial reaction step. This suggests that the rearrangements observed upon CID in the gas phase reflect the inherent reactivity of these species.

Because our experiments do not involve any hydrogen atoms, the relevance of this work to electrochemical hydrogen evolution catalyzed by molybdenum sulfide is only indirect. The mechanism for disulfide loss in Figure 6 illustrates the mobility of sulfur atoms between bridging and terminal disulfide units. Similar rearrangements were observed in our earlier studies on Mo_3_S_13_^2–^ and Mo_2_O_2_S_6_^2–^.^38,42^ These pathways seem quite robust, which suggests that they also may be involved in the reconstruction of molybdenum sulfide surface species during catalyst activation preceding HER.

## Conclusion

We studied halogenated molybdenum sulfides, specifically [Mo_3_S_7_Cl_6_]^2–^, [Mo_3_S_7_Br_6_]^2–^, and [Mo_3_S_7_I_6_]^2–^. We observed the dissociation of a halogen anion as a first fragmentation step in each case. This fragmentation channel goes from slightly endothermic via thermoneutral to slightly exothermic from Cl^–^ through Br^–^ to I^–^, respectively, according to our calculations. In addition to the overall reaction energy, however, the dianion is stabilized by a Coulomb barrier against dissociation into two negatively charged fragments, which makes [Mo_3_S_7_Br_6_]^2–^ and [Mo_3_S_7_I_6_]^2–^ metastable species. The second fragmentation step is loss of S_2_ for X = Cl, Br, while an I atom is lost from [Mo_3_S_7_I_5_]^−^, in line with the calculated barrier heights along the two competing pathways. The S_2_ loss mechanism involves a complex reconstruction of the cluster, involving S atom transfer from a bridging S_2_ unit. Similar rearrangements occur, for example, in the reaction of [Mo_3_S_7_Cl_6_]^2–^ and [Mo_3_S_7_Br_6_]^2–^ with phosphines and most likely are involved in the fragmentation of [Mo_3_S_13_]^2–^ in the gas phase. The ability to bridge any two Mo atoms in the Mo_3_ core by either one or two sulfur atoms is an intriguing chemical property, which may play an important role during catalyst activation.

## Supporting Information Available

Further experimental data, benchmark of quantum chemical methods, and Cartesian coordinates and electronic energies of all isomers (PDF)

## References

[ref1] Turner J. A. Sustainable hydrogen production. Science 2004, 305 (5686), 972–974. 10.1126/science.1103197. 15310892

[ref2] Wulf C.; Linssen J.; Zapp P. Power-to-Gas—Concepts, Demonstration, and Prospects. In Hydrogen Supply Chains; Elsevier, 2018; pp 309–345. 10.1016/B978-0-12-811197-0.00009-9.

[ref3] Walter M. G.; Warren E. L.; McKone J. R.; Boettcher S. W.; Mi Q.; Santori E. A.; Lewis N. S. Solar water splitting cells. Chem. Rev. 2010, 110 (11), 6446–6473. 10.1021/cr1002326. 21062097

[ref4] Shi Z.; Wang X.; Ge J.; Liu C.; Xing W. Fundamental understanding of the acidic oxygen evolution reaction: mechanism study and state-of-the-art catalysts. Nanoscale 2020, 12 (25), 13249–13275. 10.1039/D0NR02410D. 32568352

[ref5] Nørskov J. K.; Bligaard T.; Logadottir A.; Kitchin J. R.; Chen J. G.; Pandelov S.; Stimming U. Trends in the Exchange Current for Hydrogen Evolution. J. Electrochem. Soc. 2005, 152 (3), J23 10.1149/1.1856988.

[ref6] Vesborg P. C. K.; Seger B.; Chorkendorff I. Recent Development in Hydrogen Evolution Reaction Catalysts and Their Practical Implementation. J. Phys. Chem. Lett. 2015, 6 (6), 951–957. 10.1021/acs.jpclett.5b00306. 26262851

[ref7] Grutza M.-L.; Rajagopal A.; Streb C.; Kurz P. Hydrogen evolution catalysis by molybdenum sulfides (MoS_x_): are thiomolybdate clusters like [Mo_3_S_13_]^2-^ suitable active site models?. Sustain. Energy Fuels 2018, 10, 4228.

[ref8] Karunadasa H. I.; Montalvo E.; Sun Y.; Majda M.; Long J. R.; Chang C. J. A Molecular MoS_2_ Edge Site Mimic for Catalytic Hydrogen Generation. Science 2012, 335 (6069), 698–702. 10.1126/science.1215868. 22323816

[ref9] Kibsgaard J.; Jaramillo T. F.; Besenbacher F. Building an appropriate active-site motif into a hydrogen-evolution catalyst with thiomolybdate [Mo_3_S_13_]^2-^ clusters. Nat. Chem. 2014, 6 (3), 248–253. 10.1038/nchem.1853. 24557141

[ref10] Benck J. D.; Chen Z.; Kuritzky L. Y.; Forman A. J.; Jaramillo T. F. Amorphous Molybdenum Sulfide Catalysts for Electrochemical Hydrogen Production: Insights into the Origin of their Catalytic Activity. ACS Catal. 2012, 2 (9), 1916–1923. 10.1021/cs300451q.

[ref11] Morales-Guio C. G.; Hu X. Amorphous molybdenum sulfides as hydrogen evolution catalysts. Acc. Chem. Res. 2014, 47 (8), 2671–2681. 10.1021/ar5002022. 25065612

[ref12] Merki D.; Fierro S.; Vrubel H.; Hu X. Amorphous Molybdenum Sulfide Films as Catalysts for Electrochemical Hydrogen Production in Water. Chem. Sci. 2011, 2 (7), 1262–1267. 10.1039/C1SC00117E.

[ref13] Tran P. D.; Tran T. V.; Orio M.; Torelli S.; Truong Q. D.; Nayuki K.; Sasaki Y.; Chiam S. Y.; Yi R.; Honma I.; Barber J.; Artero V. Coordination polymer structure and revisited hydrogen evolution catalytic mechanism for amorphous molybdenum sulfide. Nat. Mater. 2016, 15, 640–646. 10.1038/nmat4588. 26974410 PMC5495159

[ref14] Chirdon D. N.; Lalisse R. F.; Sun J.; Zhang S.; Garrett B. R.; Hadad C. M.; Wu Y. [Mo_2_O_2_S_8_]^2–^ small molecule dimer as a basis for hydrogen evolution reaction (HER) catalyst materials. SN Appl. Sci. 2020, 2 (5), 889 10.1007/s42452-020-2706-3.

[ref15] Garrett B. R.; Click K. A.; Durr C. B.; Hadad C. M.; Wu Y. [MoO(S_2_)_2_L]^1-^ (L = picolinate or pyrimidine-2-carboxylate) Complexes as MoS_*x*_ Inspired Electrocatalysts for Hydrogen Production in Aqueous Solution.. J. Am. Chem. Soc. 2016, 138 (41), 13726–13731. 10.1021/jacs.6b08652. 27690413

[ref16] Garrett B. R.; Polen S. M.; Click K. A.; He M.; Huang Z.; Hadad C. M.; Wu Y. Tunable Molecular MoS_2_ Edge-Site Mimics for Catalytic Hydrogen Production. Inorg. Chem. 2016, 55 (8), 3960–3966. 10.1021/acs.inorgchem.6b00206. 27022836

[ref17] Garrett B. R.; Polen S. M.; Pimplikar M.; Hadad C. M.; Wu Y. Anion-Redox Mechanism of MoO(S_2_)_2_(2,2’-bipyridine) for Electrocatalytic Hydrogen Production. J. Am. Chem. Soc. 2017, 139 (12), 4342–4345. 10.1021/jacs.7b01350. 28296392

[ref18] Huang Z.; Luo W.; Ma L.; Yu M.; Ren X.; He M.; Polen S.; Click K.; Garrett B.; Lu J.; Amine K.; Hadad C.; Chen W.; Asthagiri A.; Wu Y. Dimeric [Mo_2_S_12_]^2-^ cluster: a molecular analogue of MoS_2_ edges for superior hydrogen-evolution electrocatalysis. Angew. Chem., Int. Ed. 2015, 54 (50), 15181–15185. 10.1002/anie.201507529. 26482571

[ref19] Dave M.; Rajagopal A.; Damm-Ruttensperger M.; Schwarz B.; Nägele F.; Daccache L.; Fantauzzi D.; Jacob T.; Streb C. Understanding homogeneous hydrogen evolution reactivity and deactivation pathways of molecular molybdenum sulfide catalysts. Sustain. Energy Fuels 2018, 2 (5), 1020–1026. 10.1039/C7SE00599G.

[ref20] Zheng H.-L.; Zhao J.-Q.; Zhang J.; Lin Q. Acid–base resistant ligand-modified molybdenum–sulfur clusters with enhanced photocatalytic activity towards hydrogen evolution. J. Mater. Chem. A 2022, 10, 7138 10.1039/D2TA00352J.

[ref21] Lang S. M.; Bernhardt T. M. Gas phase metal cluster model systems for heterogeneous catalysis. Phys. Chem. Chem. Phys. 2012, 14 (26), 9255–9269. 10.1039/c2cp40660h. 22669249

[ref22] Barwa E.; Pascher T. F.; Ončák M.; van der Linde C.; Beyer M. K. Carbon Dioxide Activation at Metal Centers: Evolution of Charge Transfer from Mg^.+^ to CO_2_ in [MgCO_2_(H_2_O)_*n*_^.+^, *n* = 0–8. Angew. Chem., Int. Ed. 2020, 59, 7467–7471. 10.1002/anie.202001292. PMC7217156 32100953

[ref23] Pascher T. F.; Ončák M.; van der Linde C.; Beyer M. K. Release of Formic Acid from Copper Formate: Hydride, Proton-Coupled Electron and Hydrogen Atom Transfer All Play their Role. ChemPhysChem 2019, 20 (11), 1420–1424. 30958610 10.1002/cphc.201900095 PMC6563433

[ref24] Pascher T. F.; Ončák M.; van der Linde C.; Beyer M. K. Decomposition of Copper Formate Clusters: Insight into Elementary Steps of Calcination and Carbon Dioxide Activation. ChemistryOpen 2019, 8, 1453–1459. 10.1002/open.201900282. 31871848 PMC6916659

[ref25] Lang S. M.; Zhou S.; Schwarz H. Tuning the oxidative power of free iron-sulfur clusters. Phys. Chem. Chem. Phys. 2017, 19 (11), 8055–8060. 10.1039/C7CP00023E. 28265613

[ref26] Kretzschmar I.; Fiedler A.; Harvey J. N.; Schröder D.; Schwarz H. Effects of sequential ligation of molybdenum cation by chalcogenides on electronic structure and gas-phase reactivity. J. Phys. Chem. A 1997, 101 (35), 6252–6264. 10.1021/jp971941+.

[ref27] Mayhall N. J.; Becher E. L. III; Chowdhury A.; Raghavachari K. Molybdenum Oxides Versus Molybdenum Sulfides: Geometric and Electronic Structures of Mo_3_X_y_^–^ (X = O, S and *y* = 6, 9) Clusters. J. Phys. Chem. A 2011, 115 (11), 2291–2296. 10.1021/jp108344k. 21366356

[ref28] Mayhall N. J.; Becher E. L. III; Chowdhury A.; Raghavachari K. Molybdenum Oxides versus Molybdenum Sulfides: Geometric and Electronic Structures of Mo_3_X_*y*_^–^ (X = O, S and *y* = 6, 9) Clusters. J. Phys. Chem. A 2011, 115 (11), 2291–2296. 10.1021/jp108344k. 21366356

[ref29] Gupta A. K.; Topolski J. E.; Nickson K. A.; Jarrold C. C.; Raghavachari K. Mo insertion into the H_2_ bond in Mo_*x*_S_*y*_^–^ + H_2_ reactions. J. Phys. Chem. A 2019, 123 (33), 7261–7269. 10.1021/acs.jpca.9b04079. 31403804

[ref30] Mason J. L.; Gupta A. K.; McMahon A. J.; Folluo C. N.; Raghavachari K.; Jarrold C. C. The striking influence of oxophilicity differences in heterometallic Mo-Mn oxide cluster reactions with water. J. Chem. Phys. 2020, 152 (5), 54301 10.1063/1.5142398. 32035442

[ref31] Waller S. E.; Jarrold C. C. *R*H and H_2_ production in reactions between *R*OH and small molybdenum oxide cluster anions. J. Phys. Chem. A 2014, 118 (37), 8493–8504. 10.1021/jp502021k. 24661103

[ref32] Topolski J. E.; Gupta A. K.; Nickson K. A.; Raghavachari K.; Jarrold C. C. Hydrogen evolution from water reactions with molybdenum sulfide cluster anions. Int. J. Mass Spectrom. 2018, 434, 193–201. 10.1016/j.ijms.2018.09.019.

[ref33] Saha A.; Raghavachari K. Hydrogen Evolution from Water Through Metal Sulfide Reactions. J. Chem. Phys. 2013, 139 (20), 204301 10.1063/1.4830096. 24289348

[ref34] Ray M.; Waller S. E.; Saha A.; Raghavachari K.; Jarrold C. C. Comparative Study of Water Reactivity with Mo_2_O_*y*_^–^ and W_2_O_y_^–^ Clusters: A Combined Experimental and Theoretical Investigation. J. Chem. Phys. 2014, 141 (10), 104310 10.1063/1.4894760. 25217919

[ref35] Ray M.; Saha A.; Raghavachari K. Hydrogen Evolution from Water Using Mo–Oxide Clusters in the Gas Phase: DFT Modeling of a Complete Catalytic Cycle Using a Mo_2_O_4_^–^/Mo_2_O_5_^–^ Cluster Couple. Phys. Chem. Chem. Phys. 2016, 18 (36), 25687–25692. 10.1039/C6CP04259G. 27711425

[ref36] Ray M.; Schaugaard R. N.; Topolski J. E.; Kafader J. O.; Raghavachari K.; Jarrold C. C. Molybdenum Oxide Cluster Anion Reactions with C_2_H_4_ and H_2_O:Cooperativity and Chemifragmentation. J. Phys. Chem. A 2018, 122 (1), 41–52. 10.1021/acs.jpca.7b10798. 29202242

[ref37] Baloglou A.; Ončák M.; van der Linde C.; Beyer M. K. Gas-phase reactivity studies of small molybdenum cluster ions with dimethyl disulfide. Top. Catal. 2018, 61 (1–2), 20–27. 10.1007/s11244-017-0864-3. 31258300 PMC6566215

[ref38] Baloglou A.; Ončák M.; Grutza M.-L.; van der Linde C.; Kurz P.; Beyer M. K. Structural properties of gas phase molybdenum sulfide clusters [Mo_3_S_13_]^2–^, [HMo_3_S_13_]^−^, and [H_3_Mo_3_S_13_]^+^ as model systems of a promising hydrogen evolution catalyst. J. Phys. Chem. C 2019, 123, 8177–8186. 10.1021/acs.jpcc.8b08324. PMC6453024 30984322

[ref39] Baloglou A.; Plattner M.; Ončák M.; Grutza M.-L.; Kurz P.; Beyer M. K. [Mo_3_S_13_]^2-^ as a Model System for Hydrogen Evolution Catalysis by MoS_x_: Probing Protonation Sites in the Gas Phase by Infrared Multiple Photon Dissociation Spectroscopy. Angew. Chem., Int. Ed. 2021, 60, 5074–5077. 10.1002/anie.202014449. PMC7986116 33332676

[ref40] Baloglou A.; Pritzi M.; Pascher T. F.; Hartmann J. C.; Grutza M.-L.; Ončák M.; Kurz P.; Beyer M. K. Proton transfer reactivity of molybdenum oxysulfide dianions [Mo_2_O_2_S_6_]^2–^ and [Mo_2_O_2_S_5_]^2-^: The role of Coulomb barriers. Int. J. Mass Spectrom. 2021, 464, 116558 10.1016/j.ijms.2021.116558.

[ref41] Plattner M.; Baloglou A.; Ončák M.; van der Linde C.; Beyer M. K. Structural Properties of Gas-Phase Molybdenum Oxide Clusters [Mo_4_O_13_]^2–^, [HMo_4_O_13_]^−^, and [CH_3_Mo_4_O_13_]^−^ Studied by Collision-Induced Dissociation. J. Am. Soc. Mass Spectrom. 2019, 30, 1946–1955. 10.1007/s13361-019-02294-4. 31420847 PMC6805806

[ref42] Pritzi M.; Pascher T. F.; Grutza M.-L.; Kurz P.; Ončák M.; Beyer M. K. Rearrangement and Decomposition Pathways of Bare and Hydrogenated Molybdenum Oxysulfides in the Gas Phase. Phys. Chem. Chem. Phys. 2022, 24, 16576 10.1039/D2CP01189A. 35775378

[ref43] Fedin V. P.; Sokolov M. N.; Mironov Y.; Kolesov B. A.; Tkachev S. V.; Fedorov V. Triangular thiocomplexes of molybdenum: reactions with halogens, hydrohalogen acids and phosphines. Inorg. Chim. Acta 1990, 167 (1), 39–45. 10.1016/S0020-1693(00)83936-5.

[ref44] Müller A.; Bhattacharyya R. G.; Pfefferkorn B. Eine einfache Darstellung der binären Metall-Schwefel-Cluster [Mo_3_S_13_]^2–^ und [Mo_2_S_12_]^2–^ aus MoO_4_^2–^ in praktisch quantitativer Ausbeute. Chem. Ber. 1979, 112 (2), 778–780. 10.1002/cber.19791120240.

[ref45] Herburger A.; van der Linde C.; Beyer M. K. Photodissociation spectroscopy of protonated leucine enkephalin. Phys. Chem. Chem. Phys. 2017, 19 (17), 10786–10795. 10.1039/C6CP08436B. 28233882

[ref46] Letzel M. Universal Mass Calculator (UMC) for Windows; University of Münster, 2014. https://www.uni-muenster.de/Chemie.oc/en/ms/downloads.html (accessed July 7, 2022).

[ref47] Grimme S.; Antony J.; Ehrlich S.; Krieg H. A consistent and accurate ab initio parametrization of density functional dispersion correction (DFT-D) for the 94 elements H-Pu. J. Chem. Phys. 2010, 132 (15), 154104 10.1063/1.3382344. 20423165

[ref48] Frisch M. J.; Trucks G. W.; Schlegel H. B.; Scuseria G. E.; Robb M. A.; Cheeseman J. R.; Scalmani G.; Barone V.; Petersson G. A.; Nakatsuji H.; Li X.; Caricato M.; Marenich A. V.; Bloino J.; Janesko B. G.; Gomperts R.; Mennucci B.; Hratchian H. P.; Ortiz J. V.; Izmaylov A. F.; Sonnenberg J. L.; Williams-Young D.; Ding F.; Lipparini F.; Egidi F.; Goings J.; Peng B.; Petrone A.; Henderson T.; Ranasinghe D.; Zakrzewski V. G.; Gao J.; Rega N.; Zheng G.; Liang W.; Hada M.; Ehara M.; Toyota K.; Fukuda R.; Hasegawa J.; Ishida M.; Nakajima T.; Honda Y.; Kitao O.; Nakai H.; Vreven T.; Throssell K.; Montgomery J. A. Jr.; Peralta J. E.; Ogliaro F.; Bearpark M. J.; Heyd J. J.; Brothers E. N.; Kudin K. N.; Staroverov V. N.; Keith T. A.; Kobayashi R.; Normand J.; Raghavachari K.; Rendell A. P.; Burant J. C.; Iyengar S. S.; Tomasi J.; Cossi M.; Millam J. M.; Klene M.; Adamo C.; Cammi R.; Ochterski J. W.; Martin R. L.; Morokuma K.; Farkas O.; Foresman J. B.; Fox D. J. Gaussian 16 Revision A.03, 2016.

[ref49] Parkes M. A.; Lockyear J. F.; Price S. D.; Schröder D.; Roithová J.; Herman Z. Selective dissociation in dication-molecule reactions. Phys. Chem. Chem. Phys. 2010, 12 (23), 6233–6243. 10.1039/b926049h. 20396821

[ref50] Beyer M.; Williams E. R.; Bondybey V. E. Unimolecular reactions of dihydrated alkaline earth metal dications M^2+^(H_2_O)_2_, M = Be, Mg, Ca, Sr, and Ba: Salt-bridge mechanism in the proton-transfer reaction M^2+^(H_2_O)_2_ -> MOH^+^ + H_3_O^+^. J. Am. Chem. Soc. 1999, 121 (7), 1565–1573. 10.1021/ja982653+. 16554906 PMC1409760

[ref51] Linstrom P. J.; Mallard W. G. NIST Chemistry WebBook, NIST Standard Reference Database Number 69. http://webbook.nist.gov/.

