# Supplementary material for: Decomposition of Halogenated Molybdenum Sulfide Dianions [Mo3S7X6]2– (X = Cl, Br, I)
Source: J Am Soc Mass Spectrom. 2022 Jul 29;33(9):1753–60. doi: 10.1021/jasms.2c00162 (PMC9460775; doi:10.1021/jasms.2c00162)
Supplement: Supplementary file 1 — js2c00162_si_001.pdf [file js2c00162_si_001.pdf]

Supporting Information

Decomposition of halogenated Molybdenum Sulfide Dianions $[Mo_3S_7X_6]^{2-}$ (X = Cl, Br, I)

Marco Pritzi,[a] Tobias F. Pascher,[a] Marie-Luise Grutza,[b] Philipp Kurz,[b] Milan987Oncák,[a,] and Martin K. Beyer[a,*]*

[a] Institut für Ionenphysik und Angewandte Physik, Universität Innsbruck, Technikerstrasse 25, 6020 Innsbruck, Austria

[b] Institut für Anorganische und Analytische Chemie, Albert-Ludwigs-Universität Freiburg, Albertstrasse 21, 79104 Freiburg, Germany

* *E-Mail:* milan.oncak@uibk.ac.at; martin.beyer@uibk.ac.at

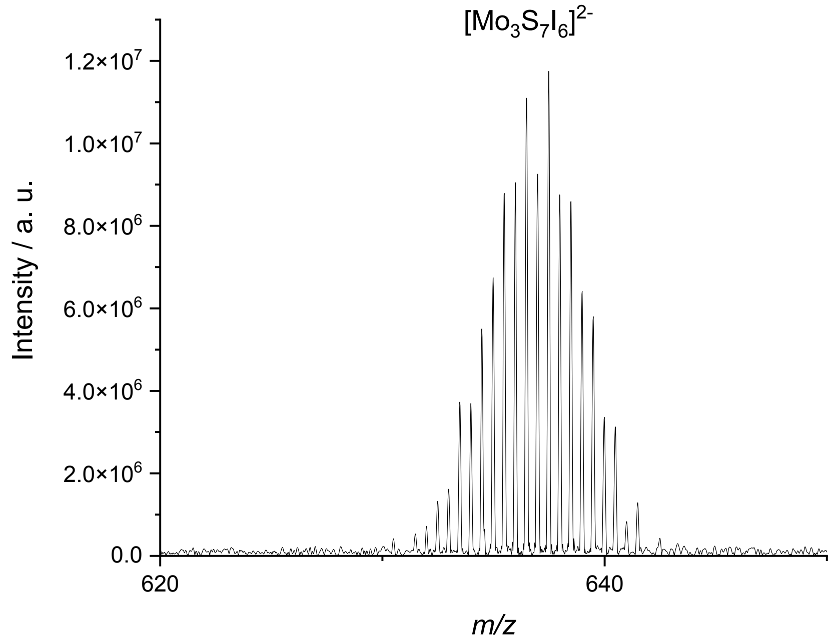

Figure S1: Mass spectrum of $[Mo_3S_7I_6]^{2-}$. The combination of molybdenum and sulfur isotopes leads to the broad distribution of isotopologues.

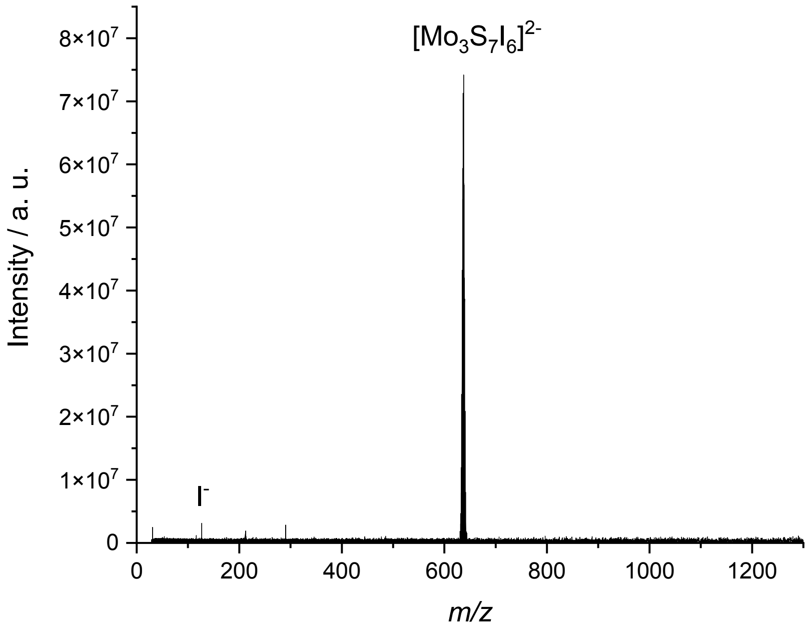

Figure S2: Accumulated mass spectrum of 200 scans with $[Mo_3S_7I_6]^{2-}$ as the precursor and a SORI power of 0 %. The iodine anion is already seen whereas $[Mo_3S_7I_5]^{2-}$ is missing.

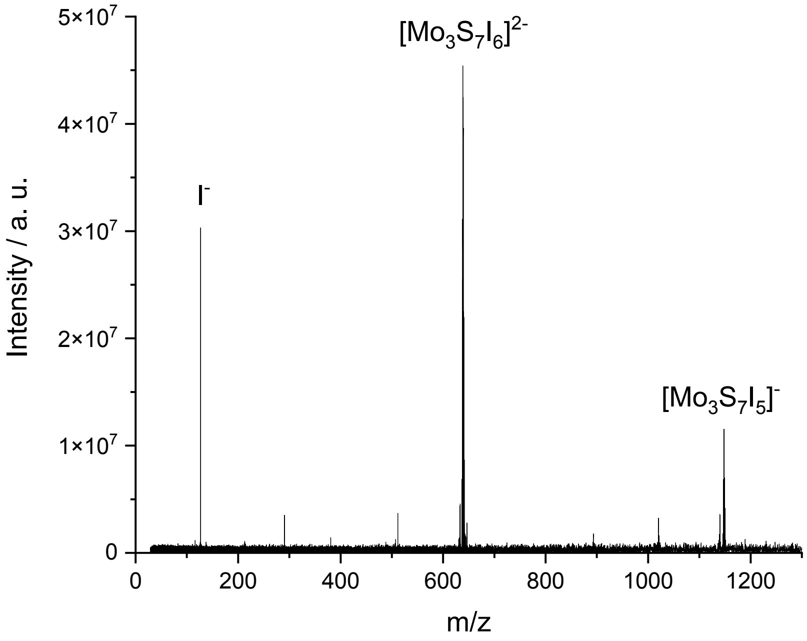

Figure S3: Accumulated mass spectrum of 200 scans with $[Mo_3S_7I_6]^{2-}$ as the precursor and a SORI power of 1.5 %. $[Mo_3S_7I_5]^{2-}$ can be identified as a first fragment.

Table S1: Benchmark of reaction energies (in eV) for B3LYP/def2TZP and ωB97XD/def2TZVP levels for reactions included in Figure 5.

Reaction	*X* = Cl		*X* = Br		*X* = I	
	B3LYP	ωB97XD	B3LYP	ωB97XD	B3LYP	ωB97XD
$[Mo_3S_7X_6]^{2-} \rightarrow [Mo_3S_7X_5]^- + X^-$	0.15	0.17	-0.05	-0.02	-0.18	-0.14
$[Mo_3S_7X_5]^- \rightarrow [Mo_3S_7X_4] + X^-$	3.12	3.13	2.76	2.79	2.44	2.48
$[Mo_3S_7X_5]^- \rightarrow [Mo_3S_7X_4]^- + X$	2.97	2.93	2.55	2.44	2.04	1.94
$[Mo_3S_7X_5]^- \rightarrow [Mo_3S_5X_5]^- + S_2$, direct	2.56	2.62	2.58	2.65	2.64	2.73
$[Mo_3S_7X_5]^- \rightarrow [Mo_3S_5X_5]^- + S_2$, indirect	1.22	1.63	1.32	1.74	1.41	1.87
$[Mo_3S_5X_5]^- \rightarrow [Mo_3S_5X_4]^- + X$	2.93	2.87	2.51	2.39	2.00	1.88

Cartesian Coordinates of optimized ions and molecules (in Å) along with the ZPE-corrected energies (in Hartree)

```
wB97XD/aug-cc-pVDZ

S2
E = -796.381127
S 0.000000 0.000000 0.942158
S -0.000000 0.000000 -0.942158

Cl-
E = -460.269794
Cl 0.000000 0.000000 0.000000

Cl
E = -460.146594
Cl 0.000000 0.000000 0.000000

Mo3S5Cl4-
E = -4037.188842
S 0.382405 0.364112 1.691366
Mo -1.344974 0.060698 0.080879
S -0.917220 1.635252 -1.551927
Mo 0.926222 1.460315 -0.285208
Cl 1.808995 3.368560 0.669837
Mo 1.091587 -1.163801 0.124414
S 2.385268 0.141719 -1.370831
Cl 2.652739 -2.861292 0.616141
S -1.097063 -2.385916 0.558387
S -0.639491 -1.757648 -1.334534
Cl -2.718127 0.848908 1.831088
Cl -3.513105 -0.354012 -1.030180

Mo3S5Cl5-, I
E = -4497.441070
Mo -0.000347 1.781438 -0.353935
S -0.000094 0.649605 1.647149
Mo 1.374346 -0.474038 0.106256
Cl 2.862994 -0.507142 1.941829
Cl 3.158361 -1.829880 -0.923174
S 0.000510 -2.506826 0.537405
S 0.000367 -1.759465 -1.351331
S 1.788431 1.101595 -1.526784
Cl -2.862690 -0.508344 1.941855
Cl -3.157596 -1.831072 -0.923186
Mo -1.374103 -0.474568 0.106252
S -1.788878 1.100917 -1.526746
Cl -0.001126 3.949842 0.401784

Mo3S5Cl5-, II
E = -4497.404649
Mo 1.010381 1.311328 -0.047145
S 0.390979 -0.000128 1.753508
Mo 1.009162 -1.312068 -0.047342
Cl 2.510681 -3.002600 0.607479
Cl 2.793330 -0.001056 -1.133664
S -1.172820 -2.491810 0.172792
S -0.739194 -1.595677 -1.606425
Cl 2.513702 2.999990 0.608361
Cl -2.741489 -0.000076 2.063443
Cl -3.526512 0.001614 -1.075046
Mo -1.340601 0.000730 0.085394
S -0.737427 1.596756 -1.606476
S -1.170328 2.493143 0.172987

Mo3S7Cl4
E = -4833.497153
S -0.332619 0.000143 1.728908
Mo 1.401809 -0.000247 0.084866
Cl 2.752383 -0.001431 2.061958
Mo -0.940674 -1.342227 -0.059363
Cl -2.036531 -3.164490 0.844325
Mo -0.939514 1.342724 -0.059807
S -1.959851 0.000284 -1.756224
S -3.048372 0.001529 -0.047099
S 0.848101 -1.653950 -1.586208
S 1.255884 -2.471213 0.214168
S 1.257361 2.470666 0.213875
S 0.849261 1.653972 -1.586782
Cl -2.033472 3.165444 0.845670
Cl 3.563249 -0.001486 -1.013685

Mo3S7Cl4-
E = -4833.627730
S -0.187257 0.279577 1.802579
Mo 1.419715 -0.070714 0.068347
Cl 2.946891 0.163458 1.940047
Mo -1.000696 -1.235488 0.219909
Cl -2.326750 -3.256877 0.170359
Mo -0.861570 1.404000 -0.119544
S -2.179789 -0.016918 -1.535026
S -3.013054 0.228322 0.313110
S 0.709233 -1.955137 -1.278349
S 1.114557 -2.460826 0.640988
S 1.419618 2.411555 -0.230397
S 0.800634 1.306702 -1.812876
Cl -1.801130 3.430562 0.640314
Cl 3.531816 -0.384200 -1.191090

Mo3S7Cl5-
E = -5293.882052
S 0.000029 0.321243 1.758133
Mo -0.000069 1.706629 -0.076079
S 1.617544 1.024285 -1.672003
S 2.490903 1.603573 0.057726
Mo 1.365170 -0.624827 0.071086
S 0.000142 -2.684588 0.289323
S -0.000072 -1.780634 -1.504225
Mo -1.365168 -0.624937 0.071180
S -1.617800 1.024462 -1.671881
S -2.490969 1.603405 0.058083
Cl 2.663227 -1.298220 2.000366
Cl 3.201927 -1.760876 -1.133055
Cl -3.202562 -1.760007 -1.132614
Cl -2.662068 -1.299771 2.000665
Cl -0.000149 3.943798 0.628028

Mo3S7Cl6--
E = -5754.158213
S -0.000027 0.000754 1.782815
Mo -0.127958 -1.575448 0.051439
S -1.670424 -0.792085 -1.589520
S -2.598107 -1.231803 0.142880
Mo -1.300162 0.898364 0.050219
S 0.232573 2.865545 0.142064
S 0.149789 1.842167 -1.590471
Mo 1.428230 0.676620 0.050211
S 1.520405 -1.051731 -1.589380
S 2.365124 -1.633902 0.143642
Cl -2.589529 1.790073 1.933916
Cl -3.080903 2.128593 -1.256321
Cl 3.384026 1.602758 -1.257125
Cl 2.845160 1.348138 1.933742
Cl -0.303443 -3.732192 -1.260511
Cl -0.254955 -3.135228 1.938596

Br-
```

E = -2574.293464
Br 0.000000 0.000000 0.000000

Br
E = -2574.171754
Br 0.000000 0.000000 0.000000

Mo3S5Br4-
E = -12493.217961
S 0.480579 0.486159 1.523898
Mo -1.228980 -0.171140 0.012820
S -1.086031 1.324943 -1.736657
Mo 0.803399 1.500354 -0.543250
Br 1.473227 3.721760 0.268924
Mo 1.350793 -1.037216 0.037067
S 2.396249 0.319462 -1.593785
Br 3.264360 -2.544990 0.628917
S -0.624998 -2.514321 0.648983
S -0.328543 -1.971259 -1.299625
Br -2.738964 0.558690 1.895682
Br -3.491623 -1.009279 -1.078204

Mo3S5Br5-, I
E = -15067.477478
Mo 0.001377 1.764096 0.665504
S 0.000391 0.843003 -1.442210
Mo -1.370830 -0.429483 -0.027271
Br -2.942346 -0.294825 -1.997805
Br -3.274728 -1.975951 0.953705
S -0.002213 -2.409780 -0.663503
S -0.001628 -1.855396 1.288035
S -1.790588 0.969596 1.755217
Br 2.941590 -0.298700 -1.998051
Br 3.271308 -1.981253 0.953926
Mo 1.369757 -0.431787 -0.027545
S 1.791573 0.966172 1.755472
Br 0.004939 4.146838 0.124308

Mo3S5Br5-, II
E = -15067.443892
Mo 0.942305 1.326202 -0.079197
S 0.274386 -0.000817 1.691534
Mo 0.942660 -1.326711 -0.079928
Br 2.478662 -3.125894 0.730400
Br 2.839354 0.000327 -1.274397
S -1.250760 -2.482598 0.083062
S -0.762562 -1.601932 -1.688832
Br 2.477310 3.127224 0.729179
Br -2.956348 0.000389 2.060264
Br -3.704232 -0.000533 -1.334121
Mo -1.401047 -0.000297 -0.021785
S -0.762533 1.602121 -1.688281
S -1.251072 2.482031 0.083880

Mo3S7Br4
E = -13289.526450
S -0.482911 0.003560 1.579089
Mo 1.286123 -0.001653 -0.015566
Br 2.668458 -0.000581 2.146801
Mo -1.051178 -1.340526 -0.221860
Br -2.256045 -3.302239 0.641258
Mo -1.046923 1.343806 -0.226055
S -2.018514 0.000181 -1.949885
S -3.152336 0.005072 -0.271120
S 0.781750 -1.657159 -1.692899
S 1.136558 -2.466734 0.120927
S 1.144445 2.464391 0.112597
S 0.786698 1.650124 -1.698547
Br -2.243689 3.308369 0.642237
Br 3.630477 -0.007243 -1.137051

Mo3S7Br4-
E = -13289.660830
S -0.300789 0.376041 1.705195
Mo 1.292401 -0.113540 -0.003374
Br 2.924363 0.175861 2.002016
Mo -1.162026 -1.186223 0.194082
Br -2.649674 -3.285961 0.285126
Mo -0.930760 1.425282 -0.274534
S -2.286984 -0.018391 -1.628291
S -3.117873 0.345291 0.199312
S 0.525694 -2.035126 -1.258052
S 0.909458 -2.456056 0.684618
S 1.383844 2.342691 -0.406096
S 0.738001 1.196451 -1.946806
Br -1.874355 3.663637 0.355978
Br 3.542367 -0.590286 -1.331046

Mo3S7Br5-
E = -15863.922384
S 0.000004 0.488849 1.623232
Mo 0.000003 1.798278 -0.267411
S 1.616881 1.043950 -1.830451
S 2.487759 1.695202 -0.126592
Mo 1.362851 -0.522970 -0.019766
S -0.000006 -2.567508 0.303728
S -0.000010 -1.756676 -1.532830
Mo -1.362856 -0.522964 -0.019761
S -1.616888 1.043965 -1.830442
S -2.487755 1.695211 -0.126575
Br 2.751198 -1.140443 2.080808
Br 3.324369 -1.794048 -1.265677
Br -3.324401 -1.794005 -1.265665
Br -2.751166 -1.140474 2.080826
Br 0.000010 4.215075 0.347144

Mo3S7Br6--
E = -18438.215276
S -1.692295 -0.000409 0.000000
Mo 0.040495 -0.788102 1.365598
S 1.675518 -1.852605 -0.000000
S -0.062103 -2.867786 -0.000000
Mo 0.040495 -0.788102 -1.365598
S -0.062368 1.434107 -2.484709
S 1.675072 0.927228 -1.604076
Mo 0.039064 1.576868 0.000000
S 1.675072 0.927228 1.604076
S -0.062368 1.434107 2.484709
Br -1.981752 -1.627062 -2.810763
Br 1.456522 -1.938885 -3.363816
Br 1.452936 3.884382 0.000000
Br -1.984954 3.245862 0.000000
Br 1.456522 -1.938885 3.363816
Br -1.981752 -1.627062 2.810763

I-
E = -297.876624
I 0.000000 0.000000 0.000000

I
E = -297.759772
I 0.000000 0.000000 0.000000

Mo3S5I4-
E = -3387.488944
S 0.570862 0.524612 1.392414
Mo -1.143965 -0.240427 -0.051973
S -1.089111 1.192982 -1.855499
Mo 0.824488 1.462015 -0.720838
I 1.496158 3.928398 0.032598
Mo 1.455939 -1.032315 -0.048990
S 2.426578 0.279921 -1.752843
I 3.578078 -2.584810 0.625120
S -0.460923 -2.535686 0.667887
S -0.227933 -2.066257 -1.306103
I -2.752966 0.539315 2.033295
I -3.590006 -1.246651 -1.178146

Mo3S5I5-, I
E = -3685.317870

Mo -0.000219 1.727243 -0.892455
S -0.000060 0.958837 1.277165
Mo 1.367431 -0.410328 -0.042726
I 3.048221 -0.138450 2.114185
I 3.428346 -2.151156 -1.015569
S 0.000366 -2.337770 0.741258
S 0.000294 -1.932346 -1.244535
S 1.796423 0.858614 -1.916069
I -3.048033 -0.139326 2.114256
I -3.427669 -2.152100 -1.015527
Mo -1.367223 -0.410693 -0.042697
S -1.796665 0.858148 -1.915998
I -0.000965 4.344257 -0.499199

Mo3S5I5-, II
E = -3685.286389
Mo 0.906812 1.343331 -0.128373
S 0.228034 0.000095 1.625545
Mo 0.906842 -1.343291 -0.128249
I 2.499161 -3.304718 0.844685
I 2.929714 -0.000004 -1.483778
S -1.297792 -2.472107 0.023911
S -0.788075 -1.610936 -1.751412
I 2.499109 3.304728 0.844652
I -3.121857 0.000090 2.168236
I -3.920389 -0.000115 -1.541180
Mo -1.428982 0.000000 -0.096959
S -0.788102 1.610776 -1.751582
S -1.297836 2.472131 0.023649

Mo3S7I4
E = -4183.799945
S -0.592129 0.000018 1.448791
Mo 1.202148 -0.000001 -0.107766
I 2.638572 0.000006 2.295353
Mo -1.126307 -1.341210 -0.366902
I -2.450457 -3.474881 0.519303
Mo -1.126293 1.341217 -0.366928
S -2.056642 -0.000011 -2.118500
S -3.222166 0.000018 -0.460852
S 0.744626 -1.659698 -1.791744
S 1.053152 -2.459205 0.035697
S 1.053168 2.459205 0.035671
S 0.744648 1.659686 -1.791765
I -2.450420 3.474897 0.519297
I 3.781635 -0.000031 -1.265457

Mo3S7I4-
E = -4183.936977
S -0.399719 0.416037 1.624476
Mo 1.197296 -0.127465 -0.061583
I 2.941914 0.214332 2.140078
Mo -1.266943 -1.169548 0.139408
I -2.900455 -3.414316 0.336219
Mo -1.002800 1.427722 -0.384857
S -2.354257 -0.035092 -1.723216
S -3.201057 0.379782 0.084446
S 0.422372 -2.070333 -1.279280
S 0.788650 -2.445908 0.675484
S 1.321837 2.313931 -0.496193
S 0.684674 1.156339 -2.031885
I -2.012628 3.884078 0.189852
I 3.647447 -0.701563 -1.473054

Mo3S7I5-
E = -4481.767875
S -0.000000 0.622927 1.501911
Mo 0.000000 1.847065 -0.447743
S 1.617109 1.020131 -1.974797
S 2.484934 1.743975 -0.298755
Mo 1.360937 -0.459058 -0.093700
S -0.000001 -2.481189 0.348249
S -0.000000 -1.779243 -1.533547
Mo -1.360937 -0.459058 -0.093700
S -1.617108 1.020131 -1.974797
S -2.484933 1.743975 -0.298756
I 2.868020 -0.993844 2.228952
I 3.487461 -1.905938 -1.385628
I -3.487462 -1.905938 -1.385628
I -2.868021 -0.993843 2.228952
I 0.000000 4.492636 0.093803

Mo3S7I6--
E = -4779.639179
S 0.000164 -1.625920 -0.000000
Mo 0.787147 0.108438 1.363832
S 1.858852 1.739547 -0.000000
S 2.864031 -0.004138 -0.000000
Mo 0.787147 0.108438 -1.363832
S -1.431918 -0.003765 -2.481277
S -0.929826 1.739649 -1.609472
Mo -1.574786 0.107542 0.000000
S -0.929826 1.739649 1.609472
S -1.431918 -0.003765 2.481277
I 1.685993 -2.101547 -2.918047
I 2.033725 1.656367 -3.524233
I -4.069848 1.655102 0.000000
I -3.369064 -2.102962 0.000000
I 2.033725 1.656367 3.524233
I 1.685993 -2.101547 2.918047

B3LYP+D3/aug-cc-pVDZ
S2
E = -796.427414
S 0.000000 -0.000000 0.951862
S -0.000000 -0.000000 -0.951862

Cl-
E = -460.288861
Cl 0.000000 0.000000 0.000000

Cl
E = -460.166979
Cl 0.000000 0.000000 0.000000

Mo3S5Cl4-
E = -4037.417829
S -0.385785 -0.388268 1.708790
Mo 1.355012 -0.023885 0.075959
S 0.970076 -1.628445 -1.566904
Mo -0.884030 -1.498407 -0.289702
Cl -1.709421 -3.447631 0.665792
Mo -1.141368 1.137808 0.141811
S -2.390496 -0.212581 -1.382065
Cl -2.725828 2.839924 0.575255
S 1.040050 2.435491 0.616113
S 0.578643 1.840174 -1.303809
Cl 2.753566 -0.840466 1.803562
Cl 3.514413 0.472078 -1.052425

Mo3S5Cl5-, I
E = -4497.692326
Mo -0.000455 1.785337 -0.361945
S -0.000108 0.661080 1.655259
Mo 1.394026 -0.477849 0.101862
Cl 2.891490 -0.486137 1.944685
Cl 3.188854 -1.843839 -0.932838
S 0.000673 -2.527520 0.583235
S 0.000491 -1.812691 -1.335804
S 1.813049 1.123803 -1.535203
Cl -2.891096 -0.487729 1.944734
Cl -3.187844 -1.845422 -0.932871
Mo -1.393701 -0.478546 0.101866
S -1.813665 1.122928 -1.535127
Cl -0.001499 3.963295 0.407312

Mo3S5Cl5-, II
E = -4497.643046
Mo 1.028665 1.266143 0.130790
S 0.203295 -0.000001 1.936878

Mo 1.026897 -1.267107 0.130442
Cl 2.324935 -3.198022 -0.429396
Cl 3.137522 -0.002017 0.287437
S -1.174511 -2.509155 0.172563
S -0.588637 -1.621819 -1.564836
Cl 2.330452 3.194321 -0.428310
Cl -2.977017 0.001198 1.885549
Cl -3.412446 0.002645 -1.337470
Mo -1.360143 0.001014 0.071546
S -0.585491 1.621583 -1.565538
S -1.171292 2.511250 0.170968

Mo3S7Cl4
E = -4833.761145
S -0.355322 -0.000228 1.731075
Mo 1.423746 0.000023 0.081974
Cl 2.753791 -0.000326 2.087532
Mo -0.949263 -1.353816 -0.072638
Cl -2.042098 -3.150793 0.908677
Mo -0.949210 1.353804 -0.072387
S -1.971524 0.000141 -1.795610
S -3.088497 0.000033 -0.086230
S 0.856721 -1.696720 -1.603748
S 1.270787 -2.494990 0.221010
S 1.270908 2.494723 0.221301
S 0.856580 1.696962 -1.603638
Cl -2.041963 3.150937 0.908711
Cl 3.595216 0.000230 -1.004828

Mo3S7Cl4-
E = -4833.888470
S -0.193419 0.306089 1.810833
Mo 1.434787 -0.106188 0.065986
Cl 2.965062 0.105684 1.954274
Mo -1.048185 -1.219998 0.236814
Cl -2.426331 -3.210046 0.170491
Mo -0.831222 1.437674 -0.136226
S -2.185437 0.026299 -1.561978
S -3.043706 0.305556 0.290962
S 0.650043 -2.009718 -1.272487
S 1.056510 -2.503800 0.665684
S 1.505208 2.399171 -0.249094
S 0.842401 1.310890 -1.845180
Cl -1.698758 3.479634 0.689703
Cl 3.546406 -0.494937 -1.191874

Mo3S7Cl5-
E = -5294.164553
S -0.000003 0.349016 -1.758588
Mo 0.000008 1.719674 0.098338
S -1.636025 1.038561 1.708975
S -2.524027 1.619325 -0.031486
Mo -1.386943 -0.631832 -0.069630
S -0.000008 -2.704736 -0.323298
S -0.000006 -1.831756 1.501475
Mo 1.386939 -0.631842 -0.069635
S 1.636036 1.038542 1.708971
S 2.524040 1.619304 -0.031489
Cl -2.681269 -1.276937 -2.028133
Cl -3.236366 -1.785895 1.118574
Cl 3.236343 -1.785930 1.118598
Cl 2.681261 -1.276933 -2.028150
Cl 0.000015 3.937219 -0.691126

Mo3S7Cl6--
E = -5754.458854
S 0.000068 0.000051 1.788035
Mo 0.963021 1.281150 0.048306
S 1.865972 -0.226935 -1.606828
S 2.886185 -0.350821 0.142029
Mo 0.628008 -1.474560 0.048438
S -1.746906 -2.324055 0.142433
S -1.129553 -1.502781 -1.606609
Mo -1.591023 0.193425 0.048445
S -0.736661 1.729466 -1.606712
S -1.139168 2.674934 0.142272
Cl 1.241290 -2.913283 1.955063
Cl 1.483252 -3.483544 -1.257275
Cl -3.758617 0.456925 -1.256338
Cl -3.143853 0.382218 1.954689
Cl 2.274742 3.026509 -1.257446
Cl 1.903230 2.531269 1.954727

Mo3S7Cl6--, TS
E = -5754.408551
mo -1.097030 -0.000132 1.046475
cl -6.720368 -0.000201 -1.975821
mo 0.890366 1.387219 -0.221499
mo 0.890594 -1.387093 -0.221567
s -0.933742 -2.520550 1.079870
s 1.117037 0.000034 1.714655
s -1.509360 -1.631243 -0.660163
s 2.712061 0.000236 -1.258431
s 0.915889 0.000112 -2.196045
s -0.934152 2.520311 1.079995
s -1.509629 1.630997 -0.660074
cl 2.584084 2.696107 0.982017
cl 1.126508 3.249093 -1.867620
cl -2.257950 -0.000279 3.103784
cl 2.584527 -2.695761 0.981885
cl 1.127038 -3.248847 -1.867780

Br-
E = -2574.264150
Br 0.000000 0.000000 0.000000

Br
E = -2574.140959
Br 0.000000 0.000000 0.000000

Mo3S5Br4-
E = -12493.252551
S 0.480983 0.521105 1.540875
Mo -1.227263 -0.215880 0.015294
S -1.143418 1.297351 -1.749166
Mo 0.748800 1.535453 -0.550220
Br 1.334743 3.801279 0.249868
Mo 1.408669 -0.991942 0.059207
S 2.393098 0.400623 -1.606430
Br 3.359351 -2.486014 0.610642
S -0.537811 -2.548575 0.722967
S -0.236990 -2.047127 -1.252583
Br -2.778610 0.545327 1.870814
Br -3.468697 -1.167292 -1.088764

Mo3S5Br5-, I
E = -15067.485743
Mo -0.002152 1.759958 0.684258
S -0.000861 0.862052 -1.444041
Mo -1.387537 -0.438125 -0.027746
Br -2.980332 -0.251537 -1.999967
Br -3.301758 -2.018692 0.949416
S 0.003610 -2.420120 -0.734516
S 0.002758 -1.920928 1.248784
S -1.816504 0.971991 1.771150
Br 2.981436 -0.245173 -1.999961
Br 3.307375 -2.010128 0.949564
Mo 1.389334 -0.434455 -0.027683
S 1.814562 0.977146 1.771072
Br -0.007925 4.160040 0.152092

Mo3S5Br5-, II
E = -15067.439277
Mo 0.947225 1.316138 -0.108348
S 0.293971 0.000196 1.687900
Mo 0.947495 -1.315919 -0.108093
Br 2.518571 -3.050946 0.802992
Br 2.829842 0.000025 -1.387993
S -1.256150 -2.507632 0.089661
S -0.777323 -1.637758 -1.710511

Br 2.518720 3.050762 0.802792
Br -2.950675 0.000744 2.099976
Br -3.758137 -0.000530 -1.314139
Mo -1.422630 -0.000098 -0.027294
S -0.777423 1.636947 -1.711196
S -1.256138 2.507810 0.088513

Mo3S7Br4
E = -13289.595969
S -0.512243 -0.000054 1.564667
Mo 1.306336 -0.000055 -0.025130
Br 2.651966 -0.000380 2.188615
Mo -1.057369 -1.352867 -0.255201
Br -2.263558 -3.291152 0.696709
Mo -1.057041 1.353044 -0.255135
S -2.026657 0.000243 -2.010483
S -3.189118 0.000423 -0.332707
S 0.800503 -1.695411 -1.722041
S 1.152845 -2.488457 0.116666
S 1.153472 2.488235 0.116259
S 0.800588 1.695060 -1.722360
Br -2.262604 3.291633 0.696988
Br 3.676163 -0.000264 -1.115754

Mo3S7Br4-
E = -13289.726769
S -0.309074 0.417351 1.702097
Mo 1.304376 -0.137763 -0.007942
Br 2.938240 0.159836 2.022741
Mo -1.203286 -1.170900 0.214105
Br -2.733666 -3.250479 0.300623
Mo -0.911374 1.452095 -0.309037
S -2.295229 -0.001822 -1.660127
S -3.149866 0.407345 0.167850
S 0.477682 -2.091053 -1.237317
S 0.865575 -2.480305 0.726879
S 1.450788 2.335927 -0.448960
S 0.769537 1.188189 -1.992752
Br -1.793464 3.713701 0.381803
Br 3.562642 -0.692610 -1.328082

Mo3S7Br5-
E = -15863.961535
S 0.000001 0.529188 1.609610
Mo 0.000002 1.808054 -0.312558
S 1.635805 1.041617 -1.883557
S 2.520876 1.707182 -0.173740
Mo 1.382891 -0.529214 -0.024881
S -0.000003 -2.582604 0.349396
S -0.000003 -1.816477 -1.521686
Mo -1.382892 -0.529210 -0.024881
S -1.635804 1.041621 -1.883556
S -2.520873 1.707186 -0.173738
Br 2.769079 -1.097711 2.119166
Br 3.363284 -1.834785 -1.245300
Br -3.363292 -1.834774 -1.245297
Br -2.769075 -1.097714 2.119169
Br 0.000004 4.221330 0.368084

Mo3S7Br6--
E = -18438.223945
S -1.695816 -0.000232 0.000000
Mo 0.043200 -0.798750 1.383548
S 1.693679 -1.880078 0.000000
S -0.057851 -2.900671 0.000000
Mo 0.043200 -0.798750 -1.383548
S -0.059545 1.450432 -2.512002
S 1.692627 0.940968 -1.628968
Mo 0.042684 1.597698 0.000000
S 1.692627 0.940968 1.628968
S -0.059545 1.450432 2.512002
Br -2.006598 -1.636143 -2.829175
Br 1.467503 -1.964733 -3.406284
Br 1.465744 3.933009 0.000000
Br -2.008135 3.267674 0.000000
Br 1.467503 -1.964733 3.406284
Br -2.006598 -1.636143 2.829175

Mo3S7Br6--, TS
E = -18438.181341
mo 0.935037 -0.000001 1.169987
br 6.889890 -0.000002 -1.423312
s 0.764748 -2.517298 1.185332
mo -0.918417 -1.383193 -0.281426
mo -0.918415 1.383193 -0.281425
s 0.764751 2.517296 1.185333
s 1.507655 1.630578 -0.490015
br -1.000354 3.375976 -2.071806
br -2.845821 2.784085 0.851174
s -1.333923 0.000000 1.617390
s -2.640017 0.000001 -1.471701
s -0.769876 0.000001 -2.248200
s 1.507653 -1.630580 -0.490016
br -2.845824 -2.784082 0.851174
br -1.000358 -3.375975 -2.071807
br 1.975596 -0.000002 3.461443

I-
E = -297.896556
I 0.000000 0.000000 0.000000

I
E = -297.778401
I 0.000000 0.000000 0.000000

Mo3S5I4-
E = -3387.723516
S 0.590246 0.545531 1.408265
Mo -1.140179 -0.270141 -0.039029
S -1.136107 1.181506 -1.853882
Mo 0.789345 1.483515 -0.726070
I 1.402994 3.984665 -0.000776
Mo 1.503046 -1.008692 -0.037143
S 2.417249 0.326093 -1.780483
I 3.650205 -2.540075 0.623398
S -0.400198 -2.560833 0.741495
S -0.167550 -2.136045 -1.258946
I -2.772588 0.567657 2.019610
I -3.587236 -1.376335 -1.178251

Mo3S5I5-, I
E = -3685.575586
Mo 0.000006 1.714120 -0.915275
S 0.000002 0.983133 1.277991
Mo 1.383818 -0.417829 -0.031728
I 3.093145 -0.062524 2.110751
I 3.460825 -2.208774 -1.000100
S -0.000010 -2.340670 0.835804
S -0.000008 -2.005263 -1.179677
S 1.820496 0.848848 -1.930208
I -3.093154 -0.062492 2.110744
I -3.460842 -2.208751 -1.000101
Mo -1.383824 -0.417819 -0.031729
S -1.820491 0.848862 -1.930210
I 0.000031 4.349063 -0.562283

Mo3S5I5-, II
E = -3685.530565
Mo 0.913899 1.332937 -0.154532
S 0.242927 0.000014 1.621679
Mo 0.913902 -1.332928 -0.154517
I 2.541911 -3.221576 0.916700
I 2.907295 -0.000002 -1.621908
S -1.302439 -2.496440 0.028354
S -0.799171 -1.646021 -1.774270
I 2.541917 3.221568 0.916701
I -3.115203 0.000009 2.217886
I -3.981269 -0.000010 -1.535423
Mo -1.448079 0.000001 -0.106581
S -0.799177 1.646007 -1.774290

S -1.302445 2.496447 0.028325

Mo3S7I4
E = -4184.068857
S -0.615152 0.000002 1.430849
Mo 1.222193 -0.000001 -0.121888
I 2.611859 0.000001 2.347560
Mo -1.133595 -1.352429 -0.399808
I -2.465455 -3.470551 0.550273
Mo -1.133593 1.352430 -0.399810
S -2.067664 -0.000000 -2.177695
S -3.257133 0.000002 -0.518641
S 0.759840 -1.696626 -1.822525
S 1.069123 -2.481539 0.027948
S 1.069126 2.481538 0.027946
S 0.759844 1.696620 -1.822527
I -2.465448 3.470555 0.550271
I 3.836063 -0.000004 -1.252301

Mo3S7I4-
E = -4184.201515
S -0.408223 0.461765 1.615446
Mo 1.206188 -0.150473 -0.067779
I 2.951447 0.212625 2.172057
Mo -1.309489 -1.152513 0.158238
I -2.990262 -3.377082 0.358235
Mo -0.984923 1.453698 -0.425971
S -2.364981 -0.022186 -1.756964
S -3.233369 0.442267 0.049296
S 0.374659 -2.125839 -1.254760
S 0.744327 -2.464623 0.721982
S 1.388027 2.305056 -0.547214
S 0.714919 1.140968 -2.082014
I -1.930647 3.937528 0.193410
I 3.672475 -0.813231 -1.475416

Mo3S7I5-
E = -4482.054996
S 0.000001 0.664741 1.479827
Mo -0.000002 1.853836 -0.502232
S 1.635478 1.006854 -2.032987
S 2.518640 1.752289 -0.355349
Mo 1.379604 -0.465267 -0.100981
S 0.000001 -2.493258 0.394206
S 0.000001 -1.839090 -1.519533
Mo -1.379603 -0.465268 -0.100981
S -1.635481 1.006852 -2.032988
S -2.518642 1.752288 -0.355350
I 2.881027 -0.938938 2.277698
I 3.531535 -1.957163 -1.369720
I -3.531533 -1.957168 -1.369719
I -2.881024 -0.938941 2.277699
I -0.000004 4.501843 0.077078

Mo3S7I6--
E = -4779.944840
S -1.633113 0.000065 -0.000000
Mo 0.108093 -0.796542 1.379716
S 1.754834 -1.885254 0.000000
S -0.002551 -2.895902 0.000000
Mo 0.108093 -0.796542 -1.379716
S -0.002870 1.448005 -2.507941
S 1.754665 0.942844 -1.632979
Mo 0.108089 1.593206 -0.000000
S 1.754665 0.942844 1.632979
S -0.002870 1.448005 2.507941
I -2.135010 -1.697181 -2.939232
I 1.684815 -2.062052 -3.571424
I 1.684841 4.124033 -0.000000
I -2.135086 3.394154 -0.000000
I 1.684815 -2.062052 3.571424
I -2.135010 -1.697181 2.939232

Mo3S7I6--, TS
E = -4779.908288
mo 0.872636 -0.000001 1.198576
i 7.018247 -0.000002 -1.219662
mo -0.937230 -1.380132 -0.303801
mo -0.937228 1.380133 -0.303800
s 1.491313 -1.631222 -0.445414
s 0.695977 -2.514501 1.207810
s 0.695982 2.514498 1.207811
s 1.491315 1.631220 -0.445414
i 1.962378 -0.000002 3.693754
i -3.061917 2.897385 0.901589
s -1.410890 0.000000 1.578487
s -2.635976 0.000002 -1.525695
s -0.751290 0.000001 -2.267774
i -3.061921 -2.897381 0.901587
i -0.967512 -3.544680 -2.268615
i -0.967507 3.544682 -2.268614

Mo3S7Cl5- Pathway

TS1
E = -5294.107634
s 1.854623 0.188172 -2.242023
mo -0.114053 1.743546 -0.124959
s 2.204797 1.658712 -0.836624
mo 1.404226 -0.527155 0.146580
s 0.046438 -2.573202 0.898291
mo -1.374430 -0.649469 0.078210
s 0.119999 -2.129029 -1.072448
s -1.290187 0.726528 -1.938103
s -2.574938 1.527563 -0.523811
cl 0.021738 3.857956 0.851671
s -0.173839 0.545281 1.750275
cl -2.879813 -0.928152 1.980920
cl 2.707636 -0.317268 2.179779
cl 3.295041 -2.006035 -0.445429
cl -3.112339 -1.954449 -1.082343

LM2
E = -5294.124965
s -2.908555 0.574213 -0.535454
s -1.431889 0.343635 -1.950682
s 2.280096 1.447331 -1.333936
s 0.829968 2.953764 -1.118453
mo 1.598264 -0.179368 0.199046
s 0.840858 -2.563910 0.556745
mo -1.048284 -1.047604 0.018295
mo -0.757072 1.653478 -0.044978
s 0.769501 -1.820206 -1.336003
s -0.127852 0.435996 1.759170
cl -1.771688 3.335744 1.214894
cl -2.317549 -1.881567 1.903670
cl -2.282709 -2.796229 -1.221477
cl 2.876508 -0.058639 2.188344
cl 3.769783 -0.943217 -0.785517

TS2
E = -5294.101613
s -2.508356 1.479891 -0.538622
s -0.703378 0.526567 -2.101118
s 1.520195 0.084698 -2.197173
s 2.057536 1.694439 -1.022751
mo 1.379356 -0.531561 0.215791
s 0.131434 -2.509090 1.154606
mo -1.372452 -0.647893 0.079450
mo -0.266630 1.757873 -0.095236
s 0.115974 -2.291337 -0.842260
s -0.124661 0.555060 1.790428
cl -0.016920 3.901494 0.808602
cl -2.939330 -0.918786 1.903103
cl -3.038407 -1.943691 -1.201799
cl 2.834663 -0.195497 2.112100
cl 3.341676 -1.839830 -0.580238

LM3
E = -5294.101563

s -2.514556 1.511272 -0.455896
s -0.606789 0.527823 -2.177778
s 1.533599 0.108801 -2.167537
s 2.110081 1.688409 -0.965271
mo 1.371546 -0.536808 0.229670
s 0.107101 -2.511087 1.145950
mo -1.388351 -0.636230 0.071267
mo -0.277279 1.743262 -0.111421
s 0.101432 -2.285542 -0.849352
s -0.131603 0.550411 1.788321
cl 0.008030 3.871961 0.824024
cl -2.948039 -0.943378 1.888456
cl -3.052371 -1.875761 -1.255416
cl 2.818221 -0.244872 2.139092
cl 3.336707 -1.830941 -0.599373

TS3
E = -5294.100512
s 0.342175 0.823123 2.368430
mo 1.417856 -0.675154 -0.059274
mo 0.347733 1.706666 0.083483
mo -1.359902 -0.526385 -0.228327
s 0.156276 0.468533 -1.806125
s -0.124801 -2.603091 -0.940392
s -0.121866 -2.167792 1.025585
s 2.564195 1.442553 0.392630
cl 3.019196 -1.788560 1.405314
cl 2.912464 -1.152999 -1.862480
cl -2.745911 -0.440225 -2.214971
cl -3.356772 -1.746190 0.656447
s -2.193057 1.766119 0.622334
s -1.698141 0.369625 2.066290
cl 0.180710 3.786771 -0.989434

LM4
E = -5294.129358
s 2.606264 0.930480 -1.260037
s 1.117728 2.433454 -1.586325
mo -0.510214 1.861893 -0.039247
s 3.102670 0.994440 0.685147
mo 1.272783 -0.623795 0.162981
mo -1.535363 -0.636539 -0.029870
s -0.180352 0.396571 1.699387
s -0.119496 -2.647855 -0.178849
s -0.000312 -1.363138 -1.759490
s -2.324212 1.207315 -1.178328
cl -3.247258 -1.853340 -1.363479
cl -2.811725 -1.370621 1.812063
cl 1.917532 -1.758476 2.247293
cl 3.096445 -2.021154 -0.836505
cl -1.000836 3.680900 1.276723

TS4
E = -5294.092181
mo 0.863104 -1.081303 0.115166
s 3.715929 0.890665 0.811422
cl 2.027096 -2.948928 -1.011743
cl 1.444649 -2.225376 2.099308
s -0.014518 0.448781 1.682806
s -1.229700 -2.460367 0.090326
mo 0.251471 1.740997 -0.200245
mo -1.743560 -0.034319 0.077855
s -1.736232 1.876822 -1.223165
cl 0.689767 3.743261 0.842051
cl -3.865381 -0.546501 -1.099831
cl -3.130374 0.015600 1.991503
s -0.766945 -1.447432 -1.627782
s 1.674245 1.212362 -1.941689
s 3.019687 -0.077872 -0.770572

LM5
E = -5294.121996
mo 0.813338 -1.051991 -0.002592
s 3.760989 1.327028 0.877181
cl 1.910219 -2.959087 -1.114050
cl 1.742332 -2.071209 2.017431
s 0.064823 0.532164 1.600754
s -1.288668 -2.378412 0.480504
mo 0.266335 1.631137 -0.408610
mo -1.788583 0.029215 0.169588
s -1.709914 1.699073 -1.456140
cl 0.896210 3.739911 0.246176
cl -4.027180 -0.510095 -0.747968
cl -3.037178 0.535043 2.110911
s -1.056137 -1.657330 -1.409634
s 1.583265 0.366810 -1.875887
s 3.179353 -0.141753 -0.252072

TS5
E = -5294.118209
s 3.231717 -0.254205 0.063431
s 1.425674 0.477193 -1.968328
mo 0.830425 -0.995953 -0.107833
mo 0.179923 1.671491 -0.494587
cl 1.937583 -2.847959 -1.310378
cl 1.934479 -2.083122 1.847977
s -1.106664 -1.640543 -1.423673
s -1.161984 -2.405700 0.462955
mo -1.785727 -0.008887 0.232306
s 0.136695 0.572290 1.538398
cl -2.906512 0.360437 2.286581
cl -4.066882 -0.615309 -0.532981
s -1.905547 1.709078 -1.330096
cl 0.944430 3.749993 0.134252
s 3.707185 1.317638 1.051823

LM6
E = -5294.122499
s 3.305114 -0.335018 0.356754
s 1.247616 0.576221 -2.059749
mo 0.861758 -0.926754 -0.214177
mo 0.078208 1.709682 -0.524516
cl 2.047993 -2.718040 -1.436219
cl 2.163982 -1.953367 1.768272
s -1.099172 -1.656796 -1.443792
s -0.996371 -2.468112 0.423233
mo -1.769132 -0.091467 0.280447
s 0.181563 0.590931 1.487485
cl -2.789728 0.160675 2.407664
cl -4.052162 -0.823194 -0.365950
s -2.068184 1.644511 -1.222392
cl 0.713139 3.847520 0.066659
s 3.642566 1.412485 1.068401

TS6
E = -5294.114758
s 4.002426 1.315236 1.029251
mo 0.686532 -1.063383 -0.160550
mo -1.805686 0.105436 0.276737
mo 0.255112 1.622087 -0.565271
s 0.212148 0.574207 1.488901
s 1.317475 0.289218 -2.022105
s -1.348351 -2.346531 0.500251
s -1.302089 -1.583897 -1.389285
cl 1.689514 -2.185822 1.795817
cl 1.218203 3.645807 -0.006806
s 3.724905 -0.437007 0.333281
s -1.871153 1.806028 -1.299968
cl -4.140060 -0.406610 -0.396096
cl -2.824560 0.606490 2.351962
cl 1.734785 -2.940453 -1.355681

LM7
E = -5294.120872
S 4.062703 1.210995 0.977017
Mo 0.519983 -1.144611 -0.017115
Mo -1.903549 0.204655 0.222762
Mo 0.319177 1.489726 -0.600635
S 0.054300 0.610990 1.517156
S 1.440230 -0.028289 -1.842574

S -1.608483 -2.236404 0.785991
S -1.500515 -1.704885 -1.188130
Cl 1.964905 -1.846907 1.735052
Cl 1.361333 3.489917 -0.093118
S 4.792562 -0.202936 -0.062308
S -1.721822 1.714212 -1.538696
Cl -4.244783 -0.182305 -0.503431
Cl -2.978396 1.021481 2.173778
Cl 1.332276 -3.241554 -1.064386

Mo3S7Br5- Pathway

TS3
E = -15863.896608
s -0.417215 0.467502 -2.614442
mo -1.441680 -0.540303 0.077290
mo -0.278308 1.716567 -0.519496
mo 1.332286 -0.516745 0.102784
s -0.047410 0.809225 1.546447
s 0.031633 -2.365872 1.213355
s -0.036927 -2.279366 -0.795729
s -2.518414 1.523856 -0.661328
br -3.286477 -1.896218 -1.183501
br -2.968720 -0.605178 2.143281
br 2.916675 -0.142898 2.135414
br 3.364388 -2.085524 -0.700544
s 2.240855 1.542444 -1.134390
s 1.614301 -0.041066 -2.311440
br 0.043114 4.095323 0.187525

LM4
E = -15863.925157
s 2.396989 1.567622 -1.501982
s 0.603914 2.651716 -1.956280
mo -0.869981 1.841353 -0.364973
s 2.825252 1.907197 0.433195
mo 1.431108 -0.120216 0.045950
mo -1.280889 -0.810474 -0.129543
s -0.238205 0.649490 1.495621
s 0.570636 -2.435087 -0.097571
s 0.404514 -1.309220 -1.790379
s -2.465001 0.684493 -1.427102
br -2.734426 -2.618605 -1.463965
br -2.451491 -1.725488 1.909455
br 2.347725 -0.943155 2.382475
br 3.708206 -1.216146 -0.926634
br -1.879716 3.711760 0.851577

TS4
E = -15863.886046
mo 1.205465 -0.704406 0.034774
s 3.464402 1.922968 0.618258
br 2.907400 -2.422674 -1.065060
br 2.073857 -1.602085 2.241154
s -0.031254 0.659549 1.501129
s -0.478621 -2.555345 0.126550
mo -0.077471 1.850112 -0.464935
mo -1.569610 -0.338201 -0.046868
s -2.034101 1.423144 -1.464312
br -0.166095 4.112629 0.473206
br -3.624085 -1.507086 -1.284122
br -3.025627 -0.535354 2.013063
s -0.270222 -1.579597 -1.658038
s 1.452610 1.558390 -2.163576
s 3.069504 0.726808 -0.910212

LM5
E = -15863.916728
mo 1.088472 -0.786960 -0.083414
s 3.561275 2.068934 0.842315
br 2.647438 -2.617573 -1.190247
br 2.197700 -1.601892 2.159401
s 0.035028 0.690162 1.439227
s -0.709673 -2.474989 0.422289
mo 0.050498 1.721343 -0.617209
mo -1.661017 -0.223169 0.023547
s -1.903646 1.383173 -1.647470
br 0.348568 4.074926 -0.027677
br -3.891831 -1.275598 -0.989739
br -3.085318 0.068352 2.093192
s -0.604052 -1.783378 -1.488378
s 1.622716 0.689072 -1.987713
s 3.270005 0.517120 -0.276232

LM7
E = -15863.914988
s -4.843029 -1.269071 -0.513696
mo -0.654900 1.148774 -0.034420
mo 1.780713 -0.175079 0.056176
mo -0.447877 -1.445120 -0.760561
s -0.120435 -0.670487 1.389537
s -1.635963 0.121721 -1.873397
s 1.485707 2.227011 0.758657
s 1.314177 1.796774 -1.232581
br -2.127939 1.807498 1.943407
br -1.498362 -3.623742 -0.333611
s -4.059387 -1.159435 1.216722
s 1.562188 -1.592459 -1.772528
br 4.252360 0.314482 -0.820937
br 3.011252 -1.121224 2.065755
br -1.572323 3.438270 -1.041289

Mo3S7I5- Pathway

TS3
E = -4481.990063
s -0.459528 0.199238 -2.759281
mo -1.450637 -0.486895 0.031563
mo -0.258395 1.661593 -0.821324
mo 1.309982 -0.525393 0.012516
s -0.007438 0.979684 1.329969
s -0.018466 -2.203864 1.343427
s -0.108108 -2.351788 -0.661003
s -2.509299 1.508675 -0.869520
i -3.517865 -2.063826 -1.116604
i -3.066648 -0.231912 2.314013
i 3.068015 0.096684 2.154768
i 3.474142 -2.372262 -0.708931
s 2.248781 1.364986 -1.426421
s 1.574254 -0.318283 -2.432337
i 0.141167 4.304727 -0.374437

LM4
E = -4482.018428
mo 1.453541 0.181016 -0.068955
s 0.350713 2.666886 -2.288775
s 2.598747 2.369851 0.184687
s 2.255352 1.839759 -1.729031
mo -1.044627 1.805436 -0.658555
s -0.312768 0.853591 1.304215
s -2.480431 0.382928 -1.621667
mo -1.142639 -0.841755 -0.202402
s 0.615409 -1.279530 -1.799495
s 0.891841 -2.221698 -0.010411
i 4.085720 -0.809484 -1.049341
i 2.499195 -0.380151 2.548760
i -2.305253 -1.793368 2.092068
i -2.458830 -3.083863 -1.530207
i -2.422443 3.767510 0.475020

TS4
E = -4481.977231
mo 1.372991 -0.362806 -0.060469
s 1.223437 1.767832 -2.337486
s 3.136962 2.589361 0.445505
s 2.982956 1.311365 -1.057588
mo -0.366853 1.880212 -0.687510
s -0.093546 0.826270 1.337192
s -2.211935 1.034101 -1.632184
mo -1.416357 -0.522741 -0.131475

s 0.085777 -1.597102 -1.680659
s 0.065251 -2.484221 0.158772
i 3.529426 -1.947143 -1.170269
i 2.420706 -1.045745 2.410620
i -2.907899 -0.912455 2.146617
i -3.367949 -2.252771 -1.423954
i -0.915666 4.329102 0.172840

LM5
E = -4482.008296
mo 1.227572 -0.582011 -0.161739
s 1.608746 0.902235 -2.090385
s 3.375946 2.515691 0.799727
s 3.271943 0.936162 -0.307121
mo -0.090563 1.772872 -0.784114
s 0.008815 0.812668 1.307519
s -2.010243 1.192388 -1.769596
mo -1.557951 -0.340962 -0.076792
s -0.328685 -1.801461 -1.550725
s -0.345281 -2.454739 0.376150
i 3.145991 -2.397936 -1.326205
i 2.456601 -1.287348 2.335815
i -3.097521 -0.208930 2.198456
i -3.833098 -1.772273 -1.188633
i -0.023298 4.358132 -0.232603

LM7
E = -4482.005026
s 4.587524 2.068097 -0.204699
mo 0.885717 -0.853857 -0.006252
mo -1.773648 -0.099723 -0.064104
mo 0.162423 1.598625 -0.848040
s -0.097257 0.838329 1.318463
s 1.683759 0.287526 -1.865746
s -0.989502 -2.355284 0.755640
s -0.835566 -1.969258 -1.244706
i 2.478706 -1.094537 2.249386
i 0.906924 4.112451 -0.414768
s 5.080967 0.228865 -0.276833
s -1.803439 1.297917 -1.918037
i -4.225958 -1.238288 -1.133983
i -3.382236 0.648635 2.052150
i 2.495159 -3.059032 -0.987740
